# Babes in the wood – a unique window into sea scorpion ontogeny

**DOI:** 10.1186/1471-2148-13-98

**Published:** 2013-05-10

**Authors:** James C Lamsdell, Paul A Selden

**Affiliations:** 1 Paleontological Institute and Department of Geology, University of Kansas, 1475 Jayhawk Boulevard, Lawrence, KS 66045, USA; 2 Palaeontology Department, Natural History Museum, Cromwell Road, London SW7, 5BD, UK

**Keywords:** Palaeozoic, Pragian, Eurypterida, *Strobilopterus*, *Syntomopterella*, *Jaekelopterus*, Cottonwood Canyon, Development, Instars, Phylogeny

## Abstract

**Background:**

Few studies on eurypterids have taken into account morphological changes that occur throughout postembryonic development. Here two species of eurypterid are described from the Pragian Beartooth Butte Formation of Cottonwood Canyon in Wyoming and included in a phylogenetic analysis. Both species comprise individuals from a number of instars, and this allows for changes that occur throughout their ontogeny to be documented, and how ontogenetically variable characters can influence phylogenetic analysis to be tested.

**Results:**

The two species of eurypterid are described as *Jaekelopterus howelli* (Kjellesvig-Waering and Størmer, 1952) and *Strobilopterus proteus* sp. nov. Phylogenetic analysis places them within the Pterygotidae and Strobilopteridae respectively, both families within the Eurypterina. *Jaekelopterus howelli* shows positive allometry of the cheliceral denticles throughout ontogeny, while a number of characteristics including prosomal appendage length, carapace shape, lateral eye position, and relative breadth all vary during the growth of *Strobilopterus proteus*.

**Conclusions:**

The ontogeny of *Strobilopterus proteus* shares much in common with that of modern xiphosurans, however certain characteristics including apparent true direct development suggest a closer affinity to arachnids. The ontogenetic development of the genital appendage also supports the hypothesis that the structure is homologous to the endopods of the trunk limbs of other arthropods. Including earlier instars in the phylogenetic analysis is shown to destabilise the retrieved topology. Therefore, coding juveniles as individual taxa in an analysis is shown to be actively detrimental and alternative ways of coding ontogenetic data into phylogenetic analyses should be explored.

## Background

Eurypterids represent a major clade of extinct chelicerate arthropods that probably represent the sister group to arachnids [1,2]. They are relatively common in Silurian and Devonian Lagerstätten, to which they are generally restricted due to their unmineralized cuticle [3], and have a total range extending from the mid-Ordovician until the end-Permian, throughout which time they exhibited a euryhaline distribution, with an increasing trend towards freshwater habitats apparent through the Carboniferous and Permian [4]. By the Middle Devonian, eurypterids had become increasingly rare, with the last of the phylogenetically basal swimming forms occurring in the Emsian Beartooth Butte Formation of Wyoming. One of the species described from that locality, *Strobilopterus princetonii* (Ruedemann, 1934), is of particular interest because juvenile specimens have been recognised that show distinct morphological differences from the adults [5].

Here, we describe new eurypterid material from an older section of the Beartooth Butte Formation at Cottonwood Canyon, Wyoming, which is Pragian in age. Two species can be recognised from the locality: the pterygotid *Jaekelopterus howelli* (Kjellesvig-Waering and Størmer, 1952) which is also known from the younger section at Beartooth Butte [6], and *Strobilopterus proteus* sp. nov. Both species are included in a broad phylogenetic analysis of the Eurypterida. Remarkably, multiple instars of both species are also recognisable at the Cottonwood Canyon locality, and these represent a unique opportunity to study the postembryonic development of extinct chelicerate species. There have been few previous studies on eurypterid ontogeny, and these have tended to rely on the same few well-sampled species and focused on changes in the dorsal carapace structures or relative length/width ratios of the carapace and opisthosoma [7-9]. *Strobilopterus proteus,* meanwhile, preserves individuals from at least four instars and exhibits previously unrecognised changes in appendage and body segment dimension and structure. Chelicerate palaeontologists have tended to neglect the influence of ontogeny when describing species [10,11] and it is important to recognise that a number of taxa may be over-split taxonomically. What is largely unknown, however, is what effect including such ontogenetic species into phylogenetic analyses would have, and so the instars of *Strobilopterus proteus* are used in a brief case-study of this possibility.

The current work comprises a complete description of both eurypterid species present at Cottonwood Canyon and a phylogenetic analysis of the Eurypterida. The ontogeny of these species is then analysed using a holomorph approach [10] in order to identify morphological trends that occur during postembryonic development and compared with the known ontogeny of other eurypterid species. Finally, the influence of including juvenile taxa in phylogenetic analysis is tested using the current analysis and material.

## Methods

### Material

The bulk of the material described herein is the result of fieldwork carried out by Robert H. Denison and Eugene S. Richardson, Jr. in 1962, and accessioned in the Field Museum of Natural History, Chicago. A single specimen was collected during fieldwork led by Hans-Peter Schultze in 1983, and is held in the University of Kansas Museum of Invertebrate Paleontology, Lawrence, Kansas. All specimens are derived from the Pragian Beartooth Butte Formation section at Cottonwood Canyon, Big Horn County, Wyoming. Photographs were taken on a Canon EOS 5D Mk II digital camera with a Canon macro EF 100 mm 1:2.8L IS USM lens with the specimens submerged in ethanol. Image processing was carried out using Adobe Photoshop CS4, and interpretive drawings were prepared for publication using Adobe Illustrator CS4, on a MacBook Pro running OS X.

### Geological settings and preservation

The Lower Devonian Beartooth Butte Formation is widespread throughout much of Wyoming and Montana; however, it is the type section in Beartooth Butte and another section in Cottonwood Canyon – both in Wyoming – that are of particular palaeontological interest. The Beartooth Butte section (Park Co., 44°57'N 109°37'W) was discovered by Erling Dorf [12], who interpreted the lithology as one of a non-marine, local channel-fill deposited in quiet, shallow, estuarine conditions, and he undertook preliminary descriptions of the abundant plant material found at the locality [13,14]. Most attention, however, has focused on the diverse fish fauna, which was described by Bryant [15-18], while low numbers of associated eurypterids were described by Ruedemann [19,20], Kjellesvig-Waering [21] and Kjellesvig-Waering and Størmer [6,22]. The eurypterid fauna was recently redescribed by Tetlie [5], with the number of confirmed eurypterid species reduced to just two: *Jaekelopterus howelli* (Kjellesvig-Waering and Størmer, 1952) and *Strobilopterus princetonii* (Ruedemann, 1934). Tetlie also suggested that *Dorfopterus angusticollis* Kjellesvig-Waering, 1955 could represent the telson of *Strobilopterus*; however the style of preservation is different to that of the other arthropods at the locality and the morphology does not bear close comparison to any other eurypterid species. The eurypterid affinities of *Dorfopterus* need to be seriously questioned.

The plant material, representing a rare extensive Lower Devonian assemblage in western North America, is also receiving renewed attention with flora from both Beartooth Butte and neighbouring Cottonwood Canyon being described [23-25]. A fish fauna has also been described from Cottonwood Canyon [26-28] although it is much less diverse than at Beartooth Butte, consisting of two species of *Protaspis* Bryant, 1933, two species of *Cardipeltis* Branson and Mehl, 1931, and a species each of *Cosmaspis* Denison, 1970 and *Lampraspis* Denison, 1970 (all heterostracans), and the dipnoan (lungfish) *Uranolophus* Denison, 1968. Three scorpions from Cottonwood Canyon have also been described, each assigned to its own monospecific genus: *Hydroscorpius denisoni* Kjellesvig-Waering, 1986, *Acanthoscorpio mucronatus* Kjellesvig-Waering, 1986 and *Branchioscorpio richardsoni* Kjellesvig-Waering, 1986. Given Kjellesvig-Waering’s propensity for over-splitting scorpion species (see Dunlop *et al.*[29] and Legg *et al.*[11]) it would perhaps be wise to re-evaluate the scorpion material; however, the suggestion that *Acanthoscorpio mucronatus* is a juvenile *Strobilopterus*[30] is not supported by new eurypterid material (unfortunately the scorpion material is not currently available for study and so its true affinities and taxonomic diversity at present remains uncertain). Notwithstanding this body of work, the most abundant component of the Cottonwood Canyon fauna, the eurypterids, have not received a systematic treatment with the exception of an isolated pterygotid ramus [31].

The Cottonwood Canyon (Big Horn Co., 44°52'N 108°02'W) section is situated in the Big Horn Mountains of northern Wyoming [32], roughly 100 km east of the type section in Beartooth Butte. The Beartooth Butte Formation at the Cottonwood Canyon section consists of long, narrow bodies of sediment with lenticular cross-sections comparable to channel fill deposits; it is underlain by the Ordovician Bighorn Dolomite and overlain by the Upper Devonian Jefferson Limestone [33]. The formation largely comprises clastic sediments deposited in a carbonate-rich context, with the fossiliferous layers at Cottonwood Canyon consisting predominantly of siltstone and shale, with dolomitised sandstone interbeds rather than the massive dolomitised limestones found at Beartooth Butte. The eurypterids at Cottonwood Canyon are preserved with the original cuticle forming a reddish-brown film over dorso-ventrally flattened impressions, while the plant material is preserved predominantly as carbonaceous compressions with rare occurrences of oxidised preservation [34]. It is possible that the eurypterid material represents moulted exuviae that became entangled with waterlogged uprooted plant material – similar associations can be found in the Lower Devonian of Alken, Germany [35-37]. The eurypterid and plant material lay on the sediment surface for some time before burial, as shown by the encrustation of microconchids on both the plant material [34] and eurypterids. Ostracodes are also present, which may have been feeding on the decaying plant matter and eurypterid cuticle.

Vertebrate biostratigraphy [38,39] indicates that the Cottonwood Canyon section is late Lochkovian to early Pragian whereas the type section at Beartooth Butte is Emsian in age. Stable oxygen and isotope data [40] indicate that the Beartooth Butte Formation was deposited in an estuarine environment, with the Cottonwood Canyon section being slightly less saline than the type section. It is interesting to note that, whereas eurypterids are common at Cottonwood Canyon where the fish are less prominent, the fauna at Beartooth Butte is clearly dominated by fish, and eurypterids are relatively scarce. This is unlikely to be due to Beartooth Butte representing a more saline environment that the eurypterids could not inhabit because eurypterids were capable of tolerating a wide range of salinities [41], and a third locality for the Beartooth Butte Formation, Half Moon Canyon, is considerably less saline than either of the other localities and appears to be totally devoid of eurypterids. One possibility is that the eurypterid population dwindled in size in the period between the deposition of the Cottonwood Canyon sediments and that of the younger sediments at Beartooth Butte, eventually going extinct before formation of the beds at Half Moon Canyon, which are Givetian in age. Eurypterid diversity did decline throughout the early and mid Devonian with the majority of swimming forms, including the clades including *Strobilopterus* and *Jaekelopterus*, going extinct prior to the Frasnian [4].

### Institutional abbreviations

FMNH, Field Museum of Natural History, Chicago, USA; KUMIP, University of Kansas Museum of Invertebrate Paleontology, Kansas, USA; PU, Princeton University Geological Museum, New Jersey, USA; YPM, Peabody Museum, Yale University, New Haven, Connecticut, USA.

### Terminology

Eurypterid terminology largely follows Tollerton [42] for morphology of the carapace, metastoma, lateral eyes, prosomal appendages, genital appendage, opisthosomal differentiation, telson, and patterns of ornamentation; however, the terminology for the ventral plate morphologies follows the revised types of Tetlie *et al.*[43]. Selden [44] is followed for prosomal structures and cuticular sculpture, as well as the labelling of the appendages, with pterygotid cheliceral denticle terminology as used by Miller [45]. Terminology for the segmentation of the genital operculum follows Waterston [46].

### Phylogenetic analysis

For the phylogenetic analysis, the matrix of Lamsdell *et al.*[47] was expanded and partially combined with the existing Stylonurina matrix [48-50] and the pterygotoid matrix of Braddy *et al.*[51], resulting in a new matrix consisting of 104 characters and 63 taxa, which can be found in the Additional file 1 along with character descriptions. All of the taxa from Lamsdell *et al.*[47] and Braddy *et al.*[51] were included along with the addition of *Laurieipterus elegans* (Laurie, 1899), *Hardieopterus macrophthalmus* (Laurie, 1892), *Kokomopterus longicaudatus* (Clarke and Ruedemann, 1912), *Drepanopterus pentlandicus* (Laurie, 1892), *Megarachne servinei* Hünicken, 1980, and *Hibbertopterus scouleri* (Hibbert, 1836) from the Stylonurina matrix so that each major stylonurine clade was represented by at least two taxa. Finally, *Jakelopterus howelli* (Kjellesvig-Waering and Størmer, 1952), *Strobilopterus proteus* sp. nov. and ‘*Erieopterus*’ *laticeps* (Schmidt, 1883) were included in order to ascertain the phylogenetic position of the taxa described herein and to resolve the affinities of ‘*Erieopterus*’ *laticeps*, which was considered by Tetlie [52] and Tetlie and Cuggy [53] to represent a dolichopterid.

The analysis was performed using TNT [54] (made available with the sponsorship of the Willi Hennig Society) employing random addition sequences followed by tree bisection-reconnection (TBR) branch swapping (the *mult* command in TNT) with 100,000 repetitions with all characters unordered and of equal weight. Jackknife [55] and Bremer support [56] values were calculated in TNT and the Consistency, Retention and Rescaled Consistency Indices were calculated in Mesquite 2.73 [57]. Nonparametric bootstrapping is often difficult with morphological data due to the limited size of the dataset [58]; however, bootstrapping with 50% resampling was performed. Jackknifing was performed using simple addition sequence and tree bisection-reconnection branch swapping, with 100,000 repetitions and 33% character deletion. The matrix and character listing can be found in Additional file 1 and has been deposited in the online MorphoBank database [59] under the project code p780 and can be accessed from http://morphobank.org/permalink/?P780.

## Results

### Systematic Palaeontology

Subphylum CHELICERATA Heymons 1901

Order EURYPTERIDA Burmeister 1843

Suborder EURYPTERINA Burmeister 1843

Family STROBILOPTERIDAE fam. nov.

### Type genus

*Strobilopterus* Ruedemann, 1935.

### Included genera

*Buffalopterus* Kjellesvig-Waering and Heubusch, 1962.

### Stratigraphical range and distribution

Middle Silurian (Wenlock) to Lower Devonian (Emsian) of Estonia, Norway and Ohio, New York and Wyoming, USA.

### Diagnosis

Eurypterina with semicircular carapace; appendage VI short, barely projecting from beneath carapace; carapace ornamentation radiating out from the lateral eyes and curving around the carapace margins; row of angular scales across the posterior of metasomal tergites.

### Genus *Strobilopterus* Ruedemann 1935

v* 1935 *Strobilopterus* Ruedemann, p. 129

v. 1961 *Syntomopterus* Kjellesvig-Waering, p. 91 [preoccupied]

*2007 Syntomopterella* Tetlie, p. 1424 [replacement name for *Syntomopterus*]

### Type species

*Pterygotus princetonii* Ruedemann, 1934, by original designation.

### Included species

*Strobilopterus laticeps* (Schmidt, 1883) [= *Dolichopterus stoermeri* Caster and Kjellesvig-Waering, 1956], *Strobilopterus richardsoni* (Kjellesvig-Waering, 1961), *Strobilopterus proteus* sp. nov.

### Stratigraphical range and distribution

Middle Silurian (Wenlock) to Lower Devonian (Emsian) of Estonia, Norway and Ohio and Wyoming, North America.

### Emended diagnosis

Large Strobilopteridae with wide semicircular carapace; lateral eyes lunate to crescentic with palpebral lobe, situated between central and centrimesial sectors; I small, no denticles; II–V small with fixed spines and serrated distal podomere margins; VI short but with powerful serrations on anterior podomere margins; VI-9 larger in later instars; metastoma almost elongate petaloid; type A genital appendage undivided and long; type B genital appendage oval; both genital appendage morphs with angular spatulae; genital operculum striate ornament marked by highly sclerotized, broad lunate scales; tergite of somite VIII reduced; preabdomen short and wide; second order opisthosomal differentiation on segments 2 to 12, especially pronounced on 7; cuticular sculpture of minute pustules, adults with narrow, elongate scales arranged across the posterior of the metasomal tergites in large individuals (emended from Tetlie [5]).

### Remarks

The new species of *Strobilopterus* described from Cottonwood Canyon herein shows the characteristic ventral and appendicular morphology of *Strobilopterus* and the diagnostic dorsal carapace structure and ornamentation of *Syntomopterella*. Kjellesvig-Waering, in a personal communication to Waterston [46], considered the Cottonwood Canyon species to be assignable to *Syntomopterella*; however, the available opisthosomal material corresponds well with the type species of *Strobilopterus*. The discovery of the *Syntomopterella*-type carapace ornamentation in a species of *Strobilopterus* renders *Syntomopterella* without any unique, defining characteristics, and the two genera are therefore synonymised herein, with *Strobilopterus* being the senior synonym. Consequently, the material of *Strobilopterus richardsoni*, and that of the other eurypterids from the Holland Quarry Shale, should be re-evaluated because a number of swimming paddles assigned to *Dolichopterus asperatus* Kjellesvig-Waering, 1961 bear close resemblance to the paddles of *Strobilopterus princetonii* and *Strobilopterus proteus*.

Larger specimens of the Cottonwood Canyon *Strobilopterus* also reveal a number of characteristics that the genus shares with *Buffalopterus*, particularly the elongate scales along the posterior metasomal tergite margins, along with the dorsal carapace ornamentation of scales angled away from the lateral eyes, and cuticular ornamentation of the sternites. The type A genital appendage of *Buffalopterus* is, however, markedly different from that of *Strobilopterus*, consisting of three segments rather than a single fused segment, and so the two genera are retained as distinct entities.

**Figure 1:**
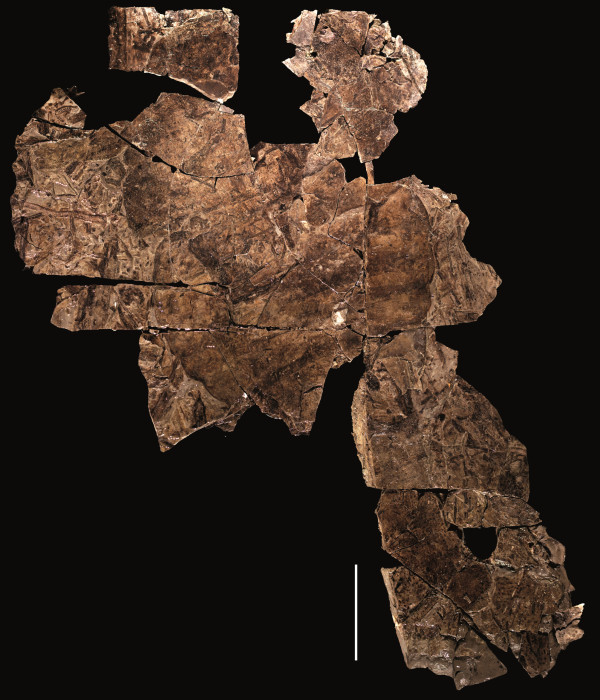
***Strobilopterus proteus.*** Holotype FMNH PE 28961. Scale bars = 50 mm.

*Strobilopterus laticeps* (Schmidt, 1883) is based on material described by Schmidt [60], Holm [61] and Størmer [62] and considered by Caster and Kjellesvig-Waering [63] to be two distinct species. The two carapaces figured by Schimdt [60] (his pl. 3, fig. 16, pl. 6, fig. 6), including the holotype, were assigned to *Erieopterus* along with a poorly preserved carapace described by Størmer ([62], fig. 1). Subsequently, a genital operculum figured by Holm ([61], pl. 4, fig. 23) was made the holotype of *Dolichopterus stoermeri* Caster and Kjellesvig-Waering, 1956, to which a metastoma figured by Holm ([61], pl. 10, fig. 10) and a swimming paddle figured by Schmidt ([60], pl. 7, fig. 9) were also assigned. The carapaces clearly belong to a strobilopterid due to their semicircular shape while the paddle is short and the type A genital operculum is a good match for *Strobilopterus* itself, possessing an elongate appendage that dorsally consists of a single unit, angular spatulae and the striate ornament on the operculum being demarcated by highly sclerotised lunate scales. Given that the dorsal and ventral material both indicates assignment to *Strobilopterus* the two species are synonymised and transferred to the genus herein.

### *Strobilopterus proteus* sp. nov.

Figures 1, 2, 3, 4, 5, 6, 7, 8, 9, 10, 11, 12, 13, 14, 15.

**Figure 2:**
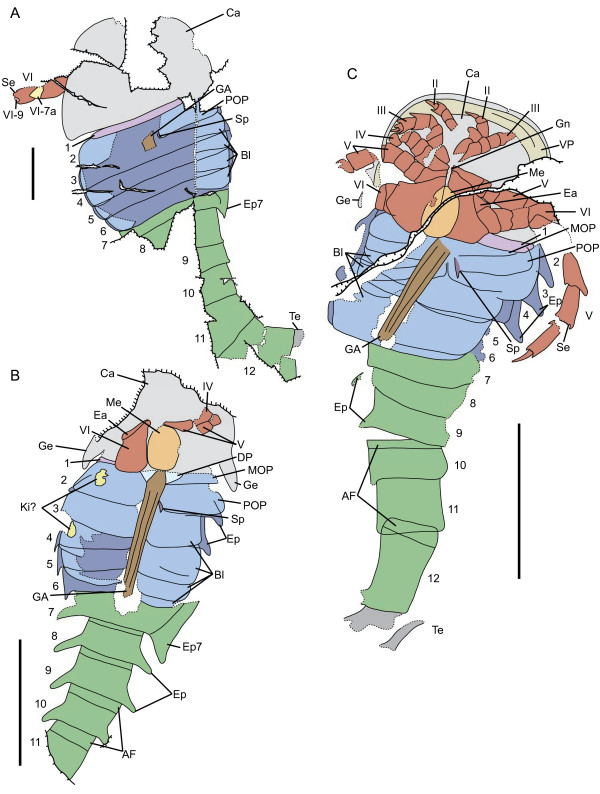
***Strobilopterus proteus.*** Interpretive drawings. **A**: Holotype, FMNH PE 28961. Scale bar = 50 mm. **B**: FMNH PE 61197. **C**: FMNH PE 61166. Scale bars = 10 mm. Specimens are colour-coded, with light grey representing the carapace, red the prosomal appendages, orange the metastoma, blue the mesosoma, green the metasoma, and dark grey the telson. The Blattfüsse are demarcated by a lighter blue, while the first opisthosomal tergite (that of somite VIII) is light purple. The genital appendage is brown, and the spatula dark purple. Dashed lines represent unnatural edges of cuticle preservation, with solid lines delineating the outline of the animal. Thick lines indicated breaks in the matrix. Abbreviations for the labels are as follows: AF, articulating facet; Bl, Blattfüsse; Ca, carapace; DP, deltoid plate; Ea, ear on coxa VI; Ep, epimera; Ep7, enlarged epimeron of opisthosomal segment 7; GA, genital appendage; Ge, carapace genal spine; Gn, gnathobase; Ki?, Kiemenplatten?; Me, metastoma; MOP, median opercular plate; POP, posterior opercular plate; Se, serrations; Sp, spatula; Te, telson; VP, prosomal ventral plates; II–VI, prosomal appendages II–VI; VI-7a, appendage VI podomere 7a; VI-9, appendage VI podomere 9; 1–12, opisthosomal segments 1–12.

**Figure 3:**
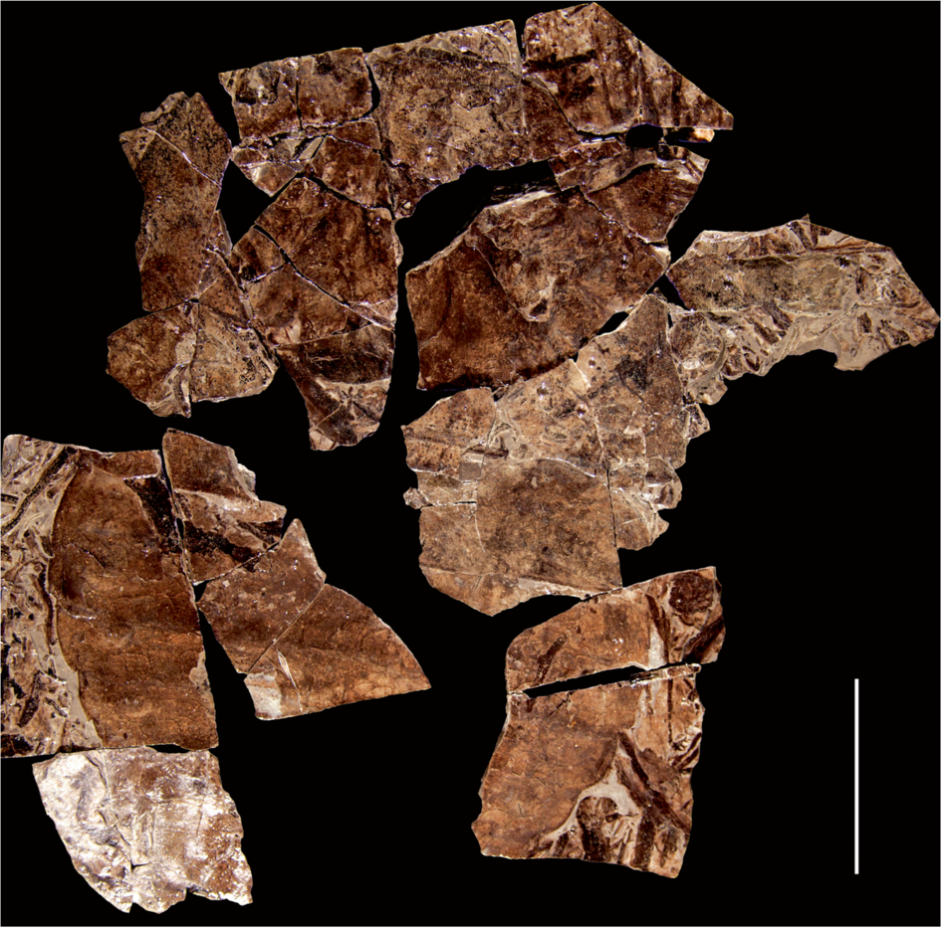
***Strobilopterus proteus.*** Counterpart to holotype FMNH PE 28961. Scale bars = 50 mm.

**Figure 4:**
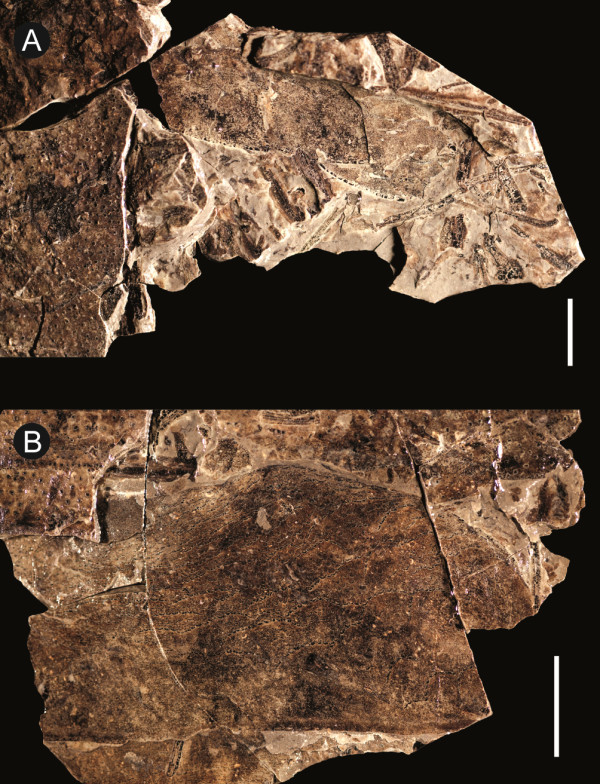
***Strobilopterus proteus.*** Details of counterpart to holotype FMNH PE 28961. **A**: prosomal appendage VI. **B**: Blattfüsse. Scale bars = 10 mm.

**Figure 5:**
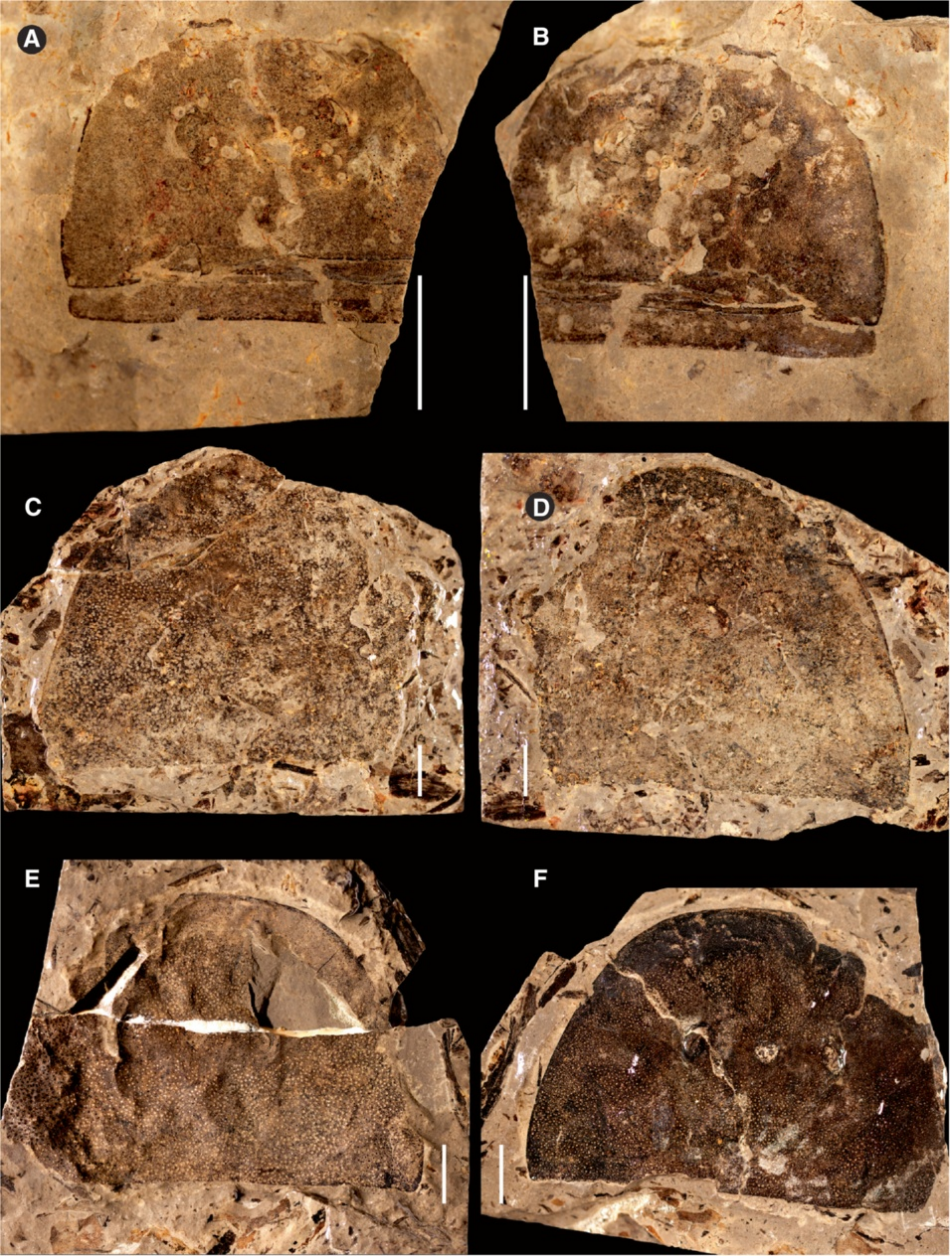
***Strobilopterus proteus.*** Carapace specimens. **A**: FMNH PE 6166. **B**: Counterpart to FMNH PE 6166. **C**: FMNH PE 61151. **D**: Counterpart to FMNH PE 61151. **E**: FMNH PE 61154. **F**: Counterpart to PE 61154. Scale bars = 10 mm.

**Figure 6:**
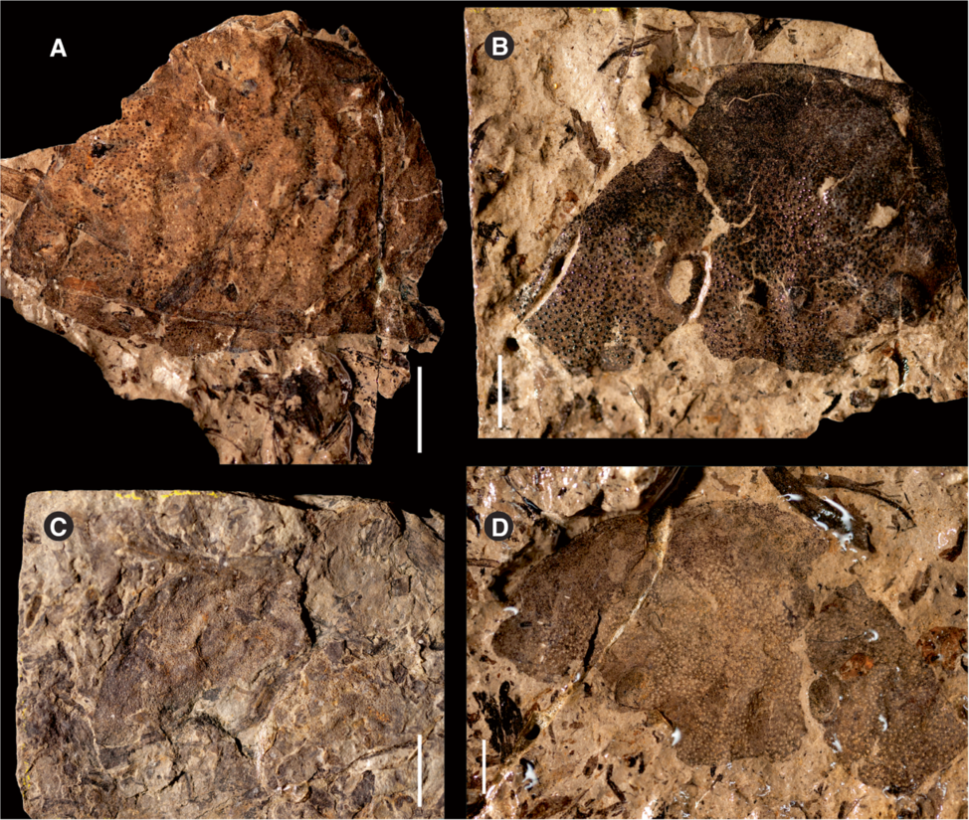
***Strobilopterus proteus.*** Carapace specimens. **A**: FMNH PE 61162. **B**: FMNH PE 61168. **C**: FMNH PE 61179. **D**: FMNH PE 7077. Scale bars = 10 mm.

**Figure 7:**
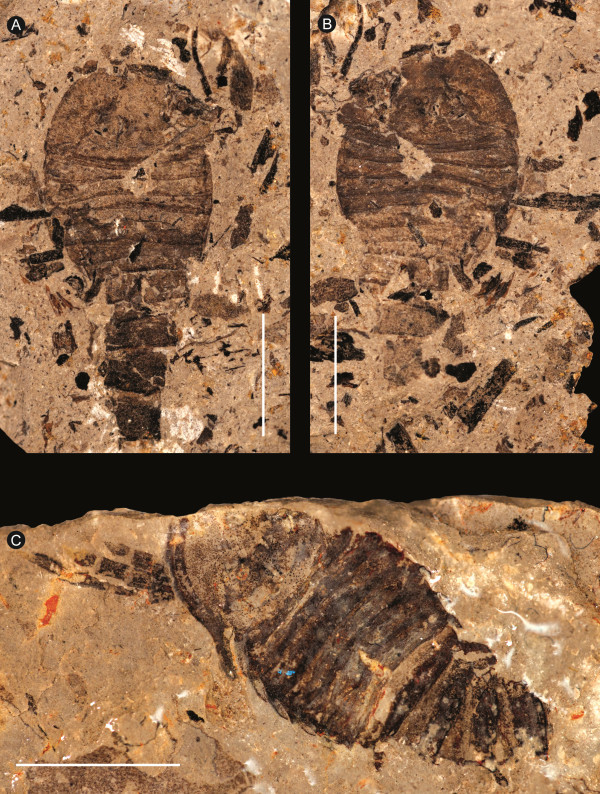
***Strobilopterus proteus.*** Juvenile specimens. **A**: FMNH PE 9236. **B**: Counterpart to FMNH PE 9236. **C**: FMNH PE 6165. Scale bars = 10 mm.

**Figure 8:**
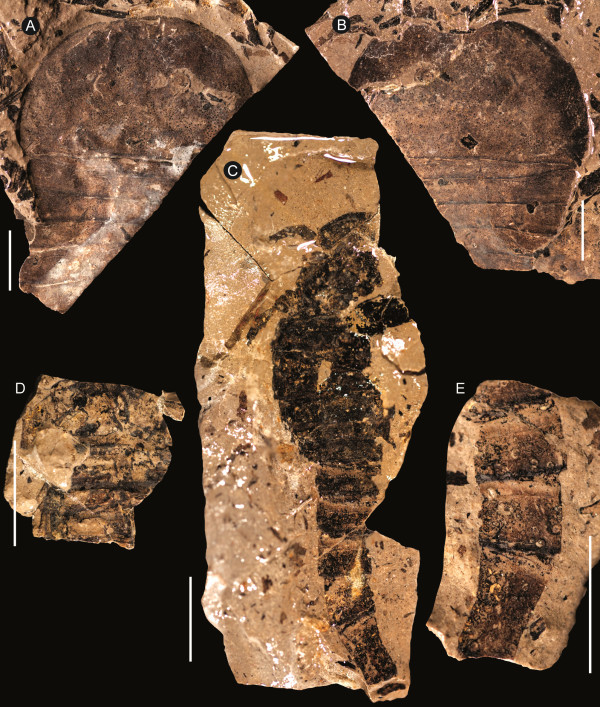
***Strobilopterus proteus.*** Juvenile specimens. **A**: FMNH PE 61166. **B**: Counterpart to FMNH PE 61166. **C**: FMNH PE 61198. **D**: Partial counterpart to FMNH PE 61198. **E**: Partial counterpart to FMNH PE 61198. Scale bars = 10 mm.

**Figure 9:**
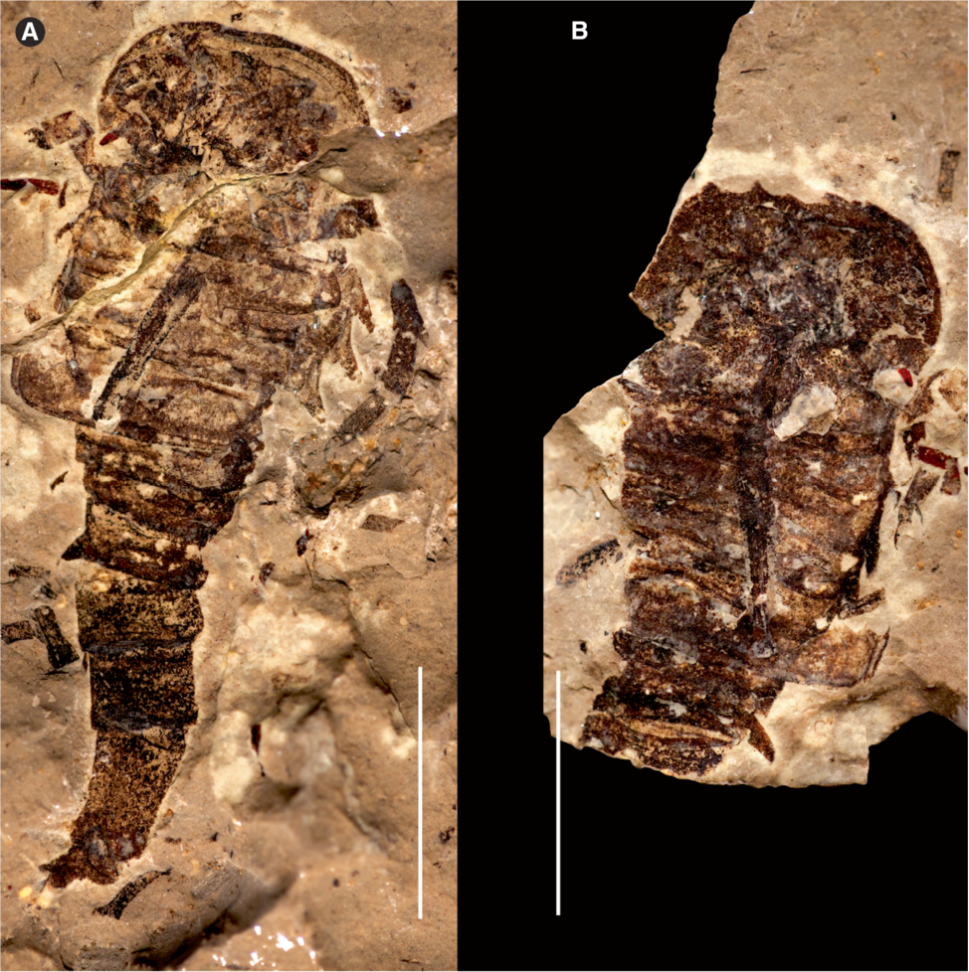
***Strobilopterus proteus.*** Juvenile specimens. **A**: FMNH PE 61197. **B**: Counterpart to FMNH PE 61197. Scale bars = 10 mm.

**Figure 10:**
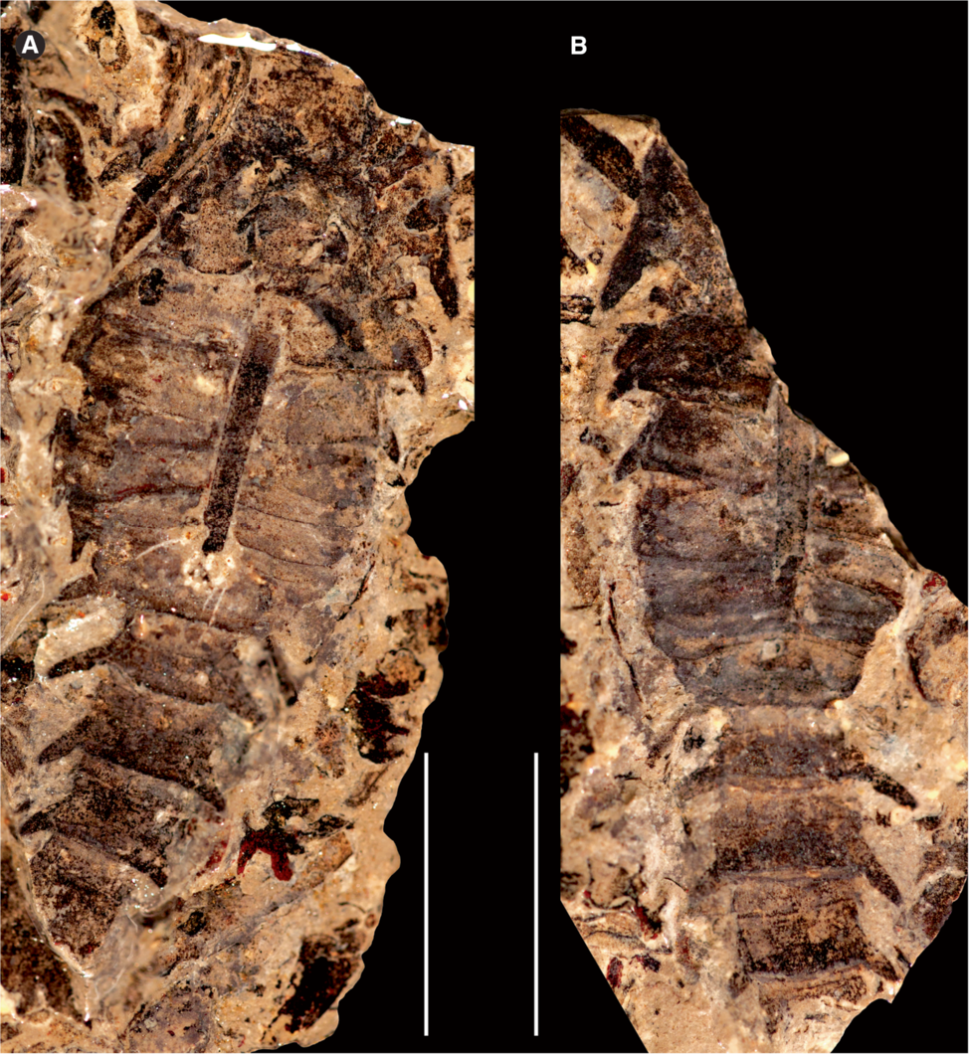
***Strobilopterus proteus.*** Juvenile specimens. **A**: FMNH PE 61199. **B**: Counterpart to FMNH PE 61199. Scale bars = 10 mm.

**Figure 11:**
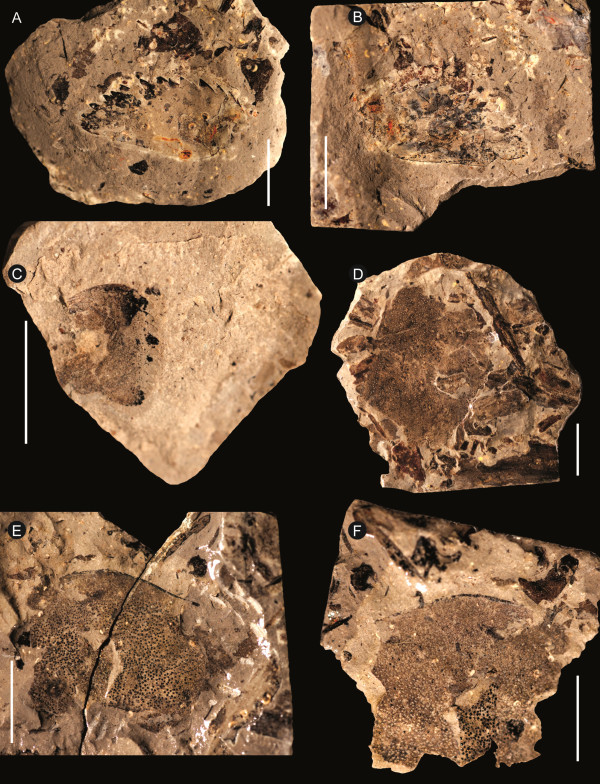
***Strobilopterus proteus.*** Coxa, paddle and carapace cuticle specimens. **A**: FMNH PE 61155, appendage VI. **B**: Counterpart to FMNH PE 61155. **C**: FMNH PE 61172, coxa. **D**: FMNH PE 61165, carapace cuticle. **E**: FMNH PE 61187, carapace cuticle showing median ocelli. **F**: Counterpart to FMNH PE 61187. Scale bars = 10 mm.

**Figure 12:**
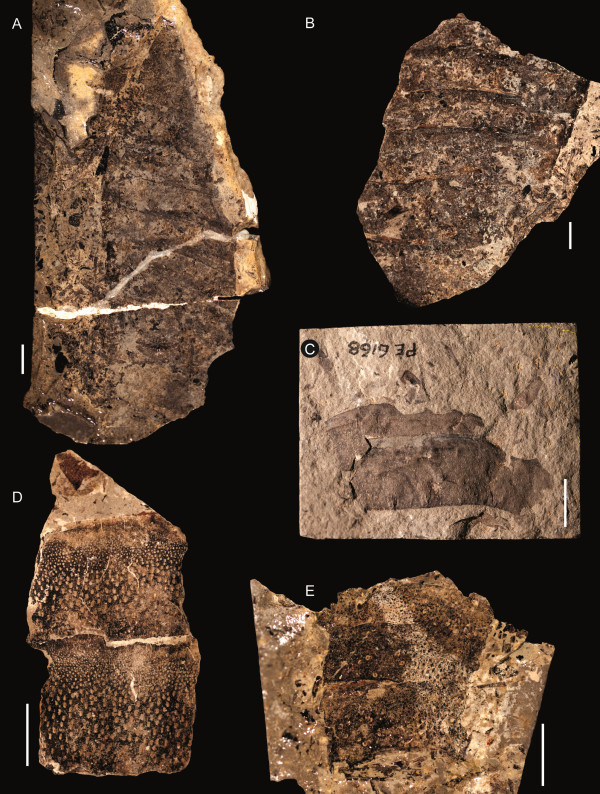
***Strobilopterus proteus.*** Opisthosomal segment specimens. **A**: FMNH PE 61191. **B**: FMNH PE 61192. **C**: FMNH PE 6168. **D**: FMNH PE 61170. **E**: FMNH PE 61185. Scale bars = 10 mm.

**Figure 13:**
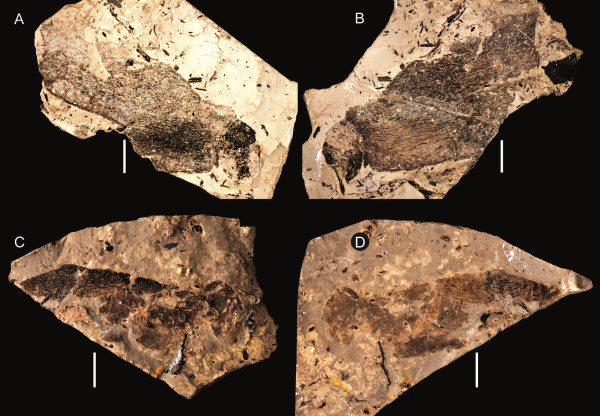
***Strobilopterus proteus.*** Type B genital operculum specimens. **A**: FMNH PE 26079. **B**: Counterpart to FMNH PE 26079. **C**: FMNH PE 61150. **D**: Counterpart to FMNH PE 61150. Scale bars = 10 mm.

**Figure 14:**
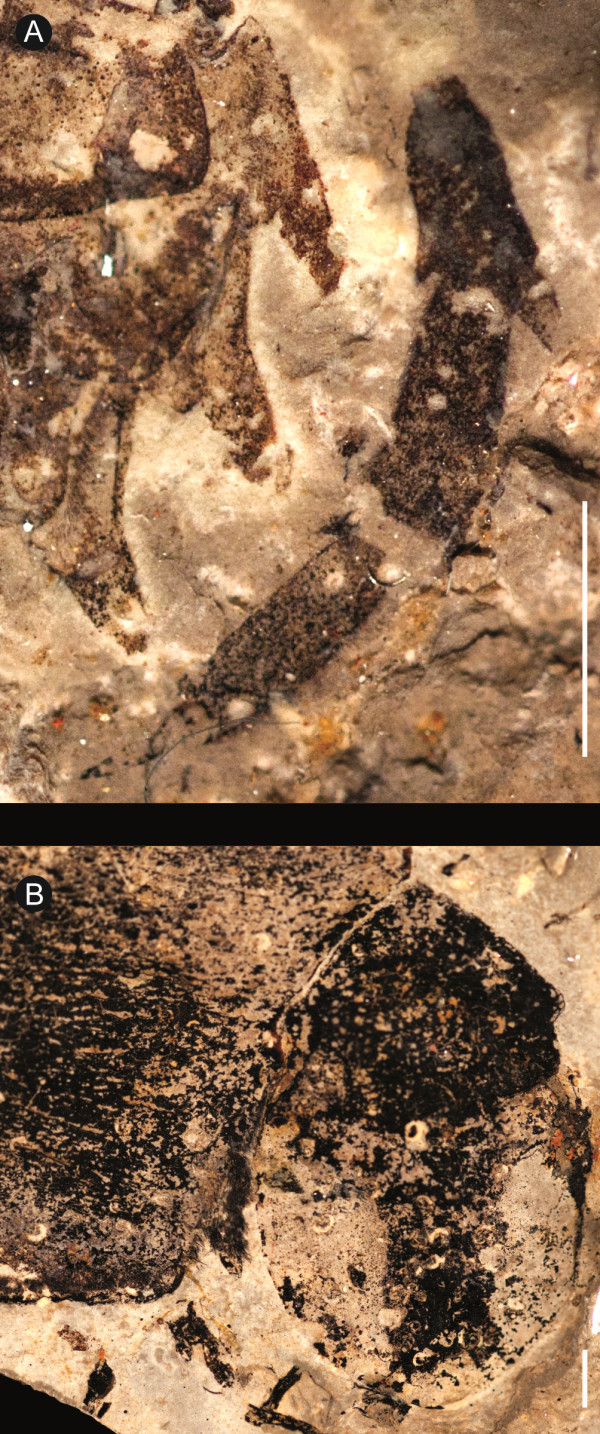
***Strobilopterus proteus.*** Closeup of opisthosomal appendage setation. **A**: FMNH PE 61197, ventral view of lateral regions of first three opisthosomal appendages, prosomal appendage V alongside. **B**: FMNH PE 26079, genital operculum. Scale bars = 2 mm.

**Figure 15:**
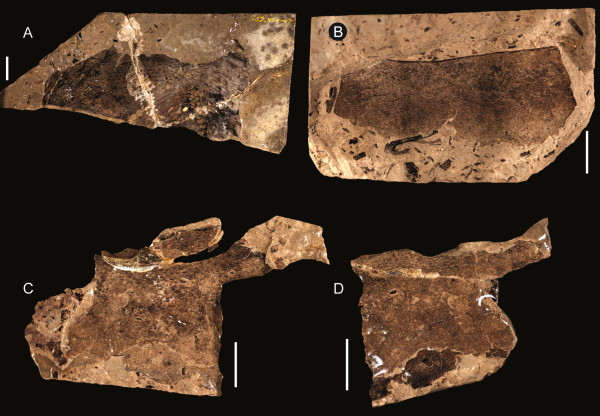
***Strobilopterus proteus.*** Cuticular specimens. **A**: FMNH PE 6167, possible genital operculum. **B**: FMNH PE 61171, Blattfüsse. **C**: FMNH PE 9242, possible Blattfüsse. **D**: Counterpart to FMNH PE 9242. Scale bars = 10 mm.

#### Etymology

Named for Proteus, a sea-god of Greek mythology and one of several deities referred to by Homer in his *Odyssey* as ‘Old Man of the Sea’, known for his ability to change shape, and origin of the adjective ‘protean’.

### Material

Holotype: FMNH PE 28961, relatively complete large individual consisting of articulated carapace, opisthosoma and proximal portion of telson, also preserving part of prosomal appendage VI. Paratypes: FMNH PE 6165–6166, PE 6168, PE 9236, PE 26079, PE 61154–61155, PE 61163, PE 61166, PE 61197–61199. Additional Material: FMNH PE 6167, PE 7077, PE 9242, PE 61150–61151, PE 61162, PE 61165, PE 61168–61172, PE 61179–61180, PE 61185, PE 61187, PE 61191–61192.

### Horizon and locality

All specimens were collected from the sole locality, the Pragian Beartooth Butte Formation section at Cottonwood Canyon, Big Horn County, Wyoming, by Robert H. Denison and Eugene S. Richardson, Jr. in 1962.

### Diagnosis

*Strobilopterus* with lateral eyes positioned on outer limits of central region; carapace cuticular ornamentation consisting of elongate pustules angling away from the lateral eyes and curving around the carapace margin; podomere VI-9 serrate, enlarged (greater than half the length of VI-8) but not longitudinally drawn-out; podomere VI-7a lacking serrations.

### Description

*Strobilopterus proteus* is known from 31 specimens which, between them, provide an almost complete view of the external morphology of the animal. Furthermore, these specimens represent a number of different instars (see discussion below) that allows for some morphological changes that occur throughout the ontogenetic development of the species to be documented.

The carapace is known from 13 specimens (Figures 1, 2, 3, 4, 5, 6, 7, 8, 9, 10), most of which also preserve details of the lateral eyes, median ocelli, and marginal rim. The carapaces range in length from 8–83 mm and from 9–133 mm in width (Table 1), with adult specimens having a length/width ratio of between 0.55 and 0.62 (Figures 1, 3, 4, 5, 6); the length/width ratio increases in the juveniles, up to a maximum of 0.83 (Figures 7, 8, 9, 10). The carapace in juveniles is, therefore, horseshoe-shaped, broadening to semicircular in adults. A marginal rim is present, extending all the way around the front and lateral edges of the carapace, and narrowing towards the posterior. This marginal rim is consistently 0.5 mm wide except in the largest specimens, where it expands in width to 1 mm; the marginal rim is, therefore, comparatively wider in juveniles compared to adults. Lateral eyes are positioned centrally in the largest specimens (e.g. FMNH PE 61151) and are crescentic, surrounding a large, moderately inflated, palpebral lobe. The lateral eyes are equivalent to 14–16% of the carapace length in adults; in the smallest juveniles they correspond to 25–30% of the carapace length and are positioned centrimesially (FMNH PE 6165), while larger adolescents have lateral eyes equal to 19–22% of the carapace length. The median ocelli are located between the lateral eyes at the carapace anteroventral midline and each circular ocellus is consistently around 1 mm in diameter so that, again, they are larger in juveniles relative to carapace size compared to adults. The ocelli in the juveniles are positioned independently on the carapace surface while in larger individuals they are located on a slight inflation (FMNH PE 61154) that appears to be cardioid in shape (FMNH PE 61154) but is not as pronounced as a true ocellar node. The greatest difference between the larger adolescent and adult specimens and the smallest juveniles is the occurrence of elongate genal spines in the latter. These are most clearly seen in FMNH PE 6165 (Figure 7C) which is dorsally preserved and shows the genal spine projecting from the posterior termination of the carapace marginal rim and extending back as far as the posterior of the second tergite. Genal spines can also be seen in the ventrally preserved specimen FMNH PE 61199 (Figure 10A) and a posterior flaring of the carapace consistent with the formation of genal spines is present in FMNH PE 61197 (Figure 9A). In adults, these genal spines are much reduced into genal facets that totally overlap the lateral margins of the first tergite (e.g. FMNH PE 6166). The carapace ornamentation consists of small, closely spaced pustules that evenly cover the dorsal surface. In both juveniles and adults, the ornamentation appears to radiate out from the lateral eyes; however, it is most noticeable in the largest individuals in which a number of pustules are somewhat elongated and clearly angled away from the lateral eyes before curving around the carapace margin (e.g. FMNH PE 61154, PE 61168).

**Table 1:** ***Strobilopterus proteus *carapace measurements**

***Specimen***	***Length***	***Width at base***	***Margin width***	***Eye length***	***Eye width***
FMNH PE 6165	8	9*	0.5	2	0.5
FMNH PE 6166	18	26*	0.5	4	1
FMNH PE 7077	39*	63*	0.5	6	2
FMNH PE 9236	10	15	0.5	3	0.5
FMNH PE 28961	83	133	1	–	–
FMNH PE 61151	56	62*	1	8	2
FMNH PE 61154	45	68*	1	7	2
FMNH PE 61162	35	51*	0.5	5	2
FMNH PE 61166	21	33*	0.5	4	1
FMNH PE 61168	36*	52*	1	6	2
FMNH PE 61179	27	29*	0.5	4	1
FMNH PE 61197	10	12	0.5	–	–
FMNH PE 61198	15	13*	0.5	–	–

All measurements in millimetres. Asterisk (*) indicates an incomplete measurement.

The ventral prosomal structures, including the appendages, are known in detail from a number of specimens, most of which are juveniles. The position of the ventral prosomal plates are visible in FMNH PE 9236 (Figure 7A), in which the plates have broken away, and FMNH PE 61197 (Figure 2C). The ventral plates appear to widen towards the posterior of the carapace while the anterior region forms a ‘triangular area’ *sensu* Størmer [36] and Lamsdell [64]. There is no evidence of a median suture and so the ventral plates are of the *Erieopterus*-type. Deep grooves anterior to the ventral plates in FMNH PE 61197 represent the sutures between the plates and the prosomal body wall that have opened up during ecdysis, as seen also in *Moselopterus* Størmer, 1974; these are distinct from the transverse sutures in Stylonurina, which occur on the ventral plates themselves. The chelicerae, which would insert close to the triangular area, are not preserved in any specimens. Elements of all the postoral prosomal appendages (II–VI) are known (Table 2), although all but appendage VI are known only from juveniles. Appendages II–V are largely homonomous in gross form, possessing an anterior spur at the distal margin of each podomere and an armature of paired, ventral, mediodistal cuticular projections. An ancillary socketed moveable spine, also located on the ventral surface of the appendage, is associated with each pair of cuticular projections. The distal margin of each podomere is denticulate. Each successive appendage increases in length, so that the second appendage is the shortest and the fifth the longest; the appendages in the smallest juveniles are also comparatively longer than in more mature individuals (e.g. FMNH PE 6165), with appendage V extending back as far as the sixth tergite in FMNH PE 61197, while in the slightly larger FMNH PE 9236 appendage II does not extend beyond the carapace margin.

**Table 2:** ***Strobilopterus proteus *prosomal appendage measurements**

***Specimen***	***Appendage***
FMNH PE 6165	**Appendage III** (podomeres 3 – 5): **3**; 1*/1. **4**; 1/1. **5**; 1*/0.5.
	**Appendage IV** (podomeres 3 – 6): **3**; 1*/1. **4**; 1/1. **5**; 2/1. **6**; 1*/0.5.
	**Appendage V** (podomeres 4 – 7): **4**; 2/1.5. **5**; 2/0.5*. **6**; 2/0.5*. **7**; 2/0.5.
FMNH PE 28961	**Appendage VI** (podomeres 7 – 8): **7**; 17*/13. **7a**; 11/8. **8**; 19*/11*.
FMNH PE 61155	**Appendage VI** (podomeres 7a – 9): **7a**; 10/5. **8**; 20/10. **9**; 6/3.
FMNH PE 61197	**Appendage II** (podomeres 5 – 7): **5**; 0.5/0.5. **6**; 0.5/0.5. **7**; 1/0.25.
	**Appendage III** (podomeres 2 – 5): **2**; 1/1. **3**; 1/1. **4**; 1/1. **5**; 1/1.
	**Appendage IV** (podomeres 1 – 4): **Coxa**; 2/1. **2**; 1/1. **3**; 1/1. **4**; 0.5*/1.
	**Appendage V** (podomeres 1 – 9): **Coxa**; 3/1.5. **2**; 1/1.5. **3**; 1/1.5. **4**; 1.5/1. **5**; 3.5/1. **6**; 2.5/1. **7**; 3/1. **8**; 3/1. **9**; 2/0.5.
	**Appendage VI** (podomeres 1 – 5): **Coxa**; 3/4. **2**; 1/2. **3**; 1/2. **4**; 1/2. **5**; 1/2.
FMNH PE 61198	**Appendage IV** (podomeres 5 – 7): **5**; 4/2. **6**; 3/1.5. **7**; 3/1.
	**Appendage VI** (podomeres 2 – 7): **2**; 2/4. **3**; 1.5/3. **4**; 2/3. **5**; 2.5/1*. **6**; 2/2*. **7**; 2*/1*.
FMNH PE 61199	**Appendage V** (podomeres 5 – 6): **5**; 3/1. **6**; 2*/1.
	**Appendage VI** (podomeres 1 – 2): **Coxa**; 3/5. **2**; 1/2.

All measurements in millimetres. Asterisk (*) indicates an incomplete measurement.

Appendage VI is known from five specimens (Figures 4, 8, 9, 10, 11), three of which are juveniles (FMNH PE 61197–61199) with the remaining two, including the holotype, being adults (FMNH PE 28961, PE 61155). The juvenile specimens preserve the proximal podomeres: the coxa (equivalent to the basipod of non-chelicerate arthropods) is expanded, with a length/width ratio of < 2.0, and has its anterior margin expanded to form an ear, although the exact shape of the ear cannot be ascertained. Podomeres VI-2–VI-5 are equal in dimension and unusually short (FMNH PE 61197), with carapace margin extending over podomere VI-6 which is still short but widens distally compared to the preceding podomeres (FMNH PE 61198). The angle between each of these podomeres is consistently 180°. VI-7 is shown in FMNH PE 61198 (Figure 8C) to be elongated and laterally expanded, although its full dimensions are not known. Podomeres VI-7–VI-9 are, however, known in detail from the adult specimens and are laterally expanded into a swimming paddle. VI-7 is at least equal in length to VI-8 and can be seen projecting out from underneath the carapace margin in the holotype (FMNH PE 28961), the VI-6/VI-7 joint being located underneath the carapace itself. The dorsal margin of VI-7 bore enlarged serrations as hinted at by the proximal region of FMNH PE 28961 (Figures 2A, 3) that shows a single serration before the dorsal margin is obscured by overhanging sediment and smaller serrations along its distal margin. The modified spine, so-called podomere 7a, is long and triangular, being about half the length of VI-8 and approximately 50% of its width. Although poorly preserved, there is no evidence on serrations along the anterior margin of VI-7a, nor are there serrations along the posterior margins of VI-7 or VI-7a. VI-8 and VI-9 are best preserved in FMNH PE 61155 (Figure 11A) which consists of both podomeres in isolation. VI-8 is longer than wide and has its dorsal margin ornamented with a series of alternating large and small serrations, although the ventral margin is devoid of ornamentation. Proximally the posterior margin of VI-8 curves anteriorly into the joint with VI-7 so that at the joint the podomere is only half its total width, which is also the width of VI-7. The gap created by this curvature of the ventral margin is covered by VI-7a. VI-9 is deeply set into VI-8, with VI-8 the ventral margin of VI-8 drawn out into an ancillary lobe, although it is unclear if this lobe articulates with the rest of VI-8 or is simply an extension of the podomere. VI-9 is large and expands distally to maintain the outline of the paddle; however, it is not distally drawn out, instead maintaining a roughly diamond-shaped outline. The antero-distal margins of VI-9 are serrated, bearing six serrations that successively decrease in size.

The metastoma is known from two juvenile specimens (FMNH PE 61197, 61199). Both are markedly longer than wide, with length/width ratios of 2.0; the FMNH PE 61197 (Figure 9) metastoma has a length of 4 mm and a width of 2 mm while the metastoma of FMNH PE 61199 (Figure 10) has a length of 6 mm and a width of 3 mm. The anterior notch is comparatively deep and the anterior shoulders rounded, while the posterior margin of the metastoma is narrow and appears rounded. In shape it is closest to elliptical (*sensu* Tollerton [42]) and is ornamented by minute scales.

Of the 15 specimens revealing dorsal details of the opisthosoma (Table 3), ten pertain to the six anterior tergites, or mesosoma (Figures 1, 3, 7, 8, 9, 10, 12). The second to sixth tergites are broadly similar, each being approximately equal in length and possessing short epimera (FMNH PE 61192). These epimera are much larger in the smallest juveniles, extending out from the anterior tergite margin into a triangular process (FMNH PE 61197). The third tergite is the broadest, measuring 137 mm in the largest specimen (FMNH PE 28961) and only 10 mm in the smallest juvenile (FMNH PE 6165). The first tergite (that of somite VIII) is however shorter than the succeeding tergites in larger individuals and is laterally reduced, lacking epimera and being overlapped by the genal regions of the carapace. The lateral portions of the second tergite also curve anteriorly, so that the carapace and second tergite occlude either side of the reduced first tergite (FMNH PE 6166, PE 28961). This is not the case in juvenile specimens, however, in which the anteriormost tergite is not differentiated and is fully laterally expressed (FMNH PE 6165, PE 9236). The cuticular ornamentation of the mesosomal tergites consists of the same small pustules as on the carapace; however, these are evenly spaced and show no differentiation in orientation. A smooth articulating facet occurs across the anterior margin of each tergite, demarcated by a row of closely spaced pustules at its posterior.

**Table 3:** ***Strobilopterus proteus *opisthosoma and telson measurements**

***Specimen***	***1***	***2***	***3***	***4***	***5***	***6***	***7***	***8***	***9***	***10***	***11***	***12***	***Telson***
FMNH PE 6165	1/9*	1/10	1/11	1/10	1/9.5	1/9	2/8	2/6	2/5	2/5	1*/3*	–	–
FMNH PE 6166	3/14*	3/25*	–	–	–	–	–	–	–	–	–	–	–
FMNH PE 6168	–	–	–	–	–	–	–	15/35*	16/44*	–	–	–	–
FMNH PE 9236	2/14	2/15	2/14	2/13	2/12	2/12	2/10	3/7	3/7	3/6	3.5/3	–	–
FMNH PE 28961	10/109	23/136	20/137	19/131	20/124	20/112	17/81*	28/74*	28/57*	28/26*	29/30*	46/43	7*/15*
FMNE PE 61163	–	–	–	–	–	–	–	–	–	19/35	22/30	30/26	–
FMNH PE 61166	3/24*	4/21*	4/18*	4/17*	4/14*	4/9*	–	–	–	–	–	–	–
FMNH PE 61170	–	–	–	–	–	–	–	–	20/26*	22/25*	–	–	–
FMNH PE 61180	–	–	–	–	–	–	–	–	–	–	–	36/31	–
FMNH PE 61185	–	–	–	–	–	–	–	11/29	11/26	13/23	–	–	–
FMNH PE 61191	–	17/40*	16/48*	16/57*	16/61*	16/63*	–	–	–	–	–	–	–
FMNH PE 61192	–	–	–	–	13/59*	12/59*	14/54*	–	–	–	–	–	–
FMNH PE 61197	–	2/13	2/14	2/12	2/10	2/9	2/8	2/6	3/6	3/5	3/4	7/4	2*/3*
FMNH PE 61198	2/8*	4/15*	4/15*	4/16*	4/17*	4/15*	5/13*	5/10	5/9	6/8	6/7	10/5	2*/4*
FMNH PE 61199	–	3/11	3/13	3/12	3/11	3/10	3/9	3/5	3/5	3/4	4/4	–	–

All measurements in millimetres. Asterisk (*) indicates an incomplete measurement.

Of the ventral mesosomal structures, both type A and type B genital appendages are known (Table 4); however, the type A morphology is only seen in juvenile specimens while only the adult type B morphology is preserved (Figure 13). The type A genital operculum is known from two specimens: FMNH PE 61197 (Figures 2C, 9) and PE 61199 (Figures 2B, 10). Neither specimen shows the sutures between the anterior, median, and opercular plates, however the right ala of FMNH PE 61199 displays portions of a striate ornament consisting of highly sclerotised semi-lunate scales alongside a dark circular structure that indicate the position of Kiemenplatten (ancillary respiratory organs; see Selden [65] and Manning and Dunlop [66]). In both specimens the centre of the genital operculum is slightly longer than its lateral portions. The type A genital appendage is long and narrow (length/width ratio ranging from 6.0–7.3), extending as far as the sixth opisthosomal segment, and is undivided with paired carinae proximally which then merge into a larger median carina. Deltoid plates are not preserved; however, angular spatulae can be seen flanking the appendage in FMNH PE 61197. The type B operculum, on the other hand, is also known from two specimens (FMNH PE 26079 and PE 61150), both of which are disarticulated and consist of an isolated type B genital appendage with a single associated ala. The most striking feature of the operculum is the striate ornament of highly sclerotized semi-lunate scales that extends laterally across the ala; these are also seen on the genital operculum of the holotype (FMNH PE 28961), although the genital appendage itself is not preserved. The genital operculum bears a clear suture dividing the median and posterior opercular plates (FMNH PE 26079) which each comprise approximately 50% of the length of the operculum. A strip of lightly coloured cuticle anterior to the main operculum near the genital appendage may represent the remnants of the anterior opercular plate. The type B genital appendage itself is oval and short, having a length/width ratio of around 1.6 and only barely projecting beyond the posterior margins of the operculum. The central portion of the appendage appears more highly sclerotised than the lateral regions, while anteriorly it is hastate where it inserts on the operculum. Triangular deltoid plates are faintly preserved either side of the hastate region. An angular spatula is preserved alongside the genital appendage in FMNH PE 26079 (Figure 13A) and is covered in short, dense setation. The internal margin of the operculum alongside the appendage also bears short bristles (Figure 14). These bristles can also be seen preserved in the post-genital opercula (Blattfüsse) of FMNH PE 61197 and PE 61199, where they form a fringe at the distal margins, and in the holotype FMNH PE 28961 (Figures 1, 3). The Blattfüsse of these specimens are medially fused with the exception of the first (i.e. that of the third opisthosomal tergite) and are ornamented with fine pustules and small scales (Figure 15). An isolated Blattfüsse of a larger individual (FMNH PE 61171) shows ornamentation similar to that of the genital operculum consisting of striations formed by highly sclerotised semi-lunate scales, suggesting this ornamentation develops in later instars.

**Table 4:** ***Strobilopterus proteus *genital operculum measurements**

***Specimen***	***Type***	***Length (centre)***	***Length (lateral)***	***Width***	***Appendage Length***	***Appendage Width***
FMNH PE 26079	B	25*	44*	108*	23	14
FMNH PE 61150	B	20*	24*	67*	18*	12
FMNH PE 61197	A	3	2	11.5	9	1.5
FMNH PE 61199	A	3	2	13	11	1.5

All measurements in millimetres. Asterisk (*) indicates an incomplete measurement.

**Figure 16:**
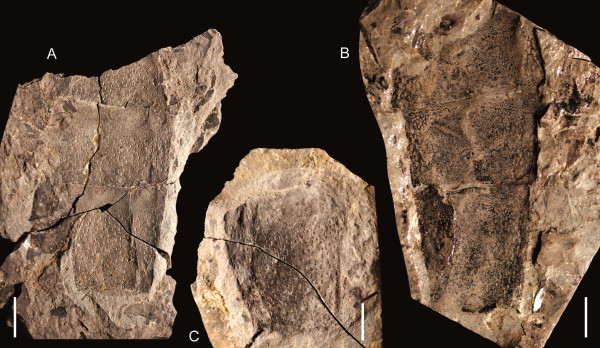
***Strobilopterus proteus.*** Metasomal segment specimens. **A**: FMNH PE 61163. **B**: Counterpart to FMNH PE 61163. **C**: FMNH PE 61180. Scale bars = 10 mm.

Aspects of the metasoma (comprising the six posterior opisthosomal segments) are known from 12 specimens, representing both the juvenile and adult morphology (Figures 1, 3, 7, 8, 9, 10, 16). The first metasomal segment (the seventh tergite) is differentiated from the rest, being of similar breadth to the mesosomal tergites and possessing large angular epimera (FMNH PE 28961). There is a sudden constriction between the seventh and eighth tergites, marking the differentiation into the preabdominal and postabdominal non-functional pseudotagmata (*sensu* Lamsdell [2]), with segments 8 to 12 narrowing evenly thereafter. These segments also bear short epimera (FMNH PE 61163), as in the mesosomal segments, while in the smallest juveniles these epimera are again enlarged, appearing peg-like and projecting from the segments at a consistent 120° angle (FMNH PE 6165, PE 61197, PE 61199). The length of the first five metasomal segments tends not to vary, while the length of segment 12 (the pretelson) is increased. The degree of pretelson elongation is comparatively greater in the juvenile specimens which have a pretelson length/width ratio of 1.7–2.0 compared to that of 1.0–1.2 in larger, adult specimens. The ornament of these metasomal segments is uniform, however, consisting of small pustules that not only decrease in density towards the posterior of the segment but also increase in size and become asymmetrical, eventually forming narrow lunate scales (FMNH PE 61163, 61170). The anterior margin of the segments comprises a smooth articulating facet with a row of dense pustules along its posterior margin (FMNH PE 61180, PE 61185). The largest specimens also possess an ornamentation of six large, acicular scales across their posterior margin (e.g. FMNH PE 28961) that are themselves covered in the regular cuticular ornamentation (FMNH PE 6168). The telson, however, is not preserved in detail on any specimen, being consistently broken off a few millimetres posterior to its articulation with the pretelson in those specimens where it is visible. A long, strait structure preserved alongside the pretelson of FMNH PE 61197 (Figure 9) probably represents a portion of the disarticulated telson, however this is still only a fragment and no further details of its morphology are available.

### Remarks

*Strobilopterus proteus* exhibits clear characteristics supporting its assignment to the genus *Strobilopterus*, specifically the morphology of the carapace and lateral eyes, the pronounced epimera on the seventh opisthosomal tergite, the cuticular ornament consisting of fine pustules with a striate ornament of highly sclerotised scales on the genital operculum and, particularly, the distinctive morphology of appendage VI. Despite the morphological disparity between the smallest juveniles and the adult specimens, both possess the pustular cuticular ornamentation and pronounced epimera on tergite seven. Furthermore, the type A genital appendage and morphology of prosomal appendages II–V, which are known only from juvenile specimens of *Strobilopterus proteus*, correspond well to those structures in *Strobilopterus princetonii*. The type A genital appendage in FMNH PE 61197 and PE 61199 is identical in morphology to that of the *Strobilopterus princetonii* holotype, YPM 204947, while the anterior prosomal limbs in FMNH PE 61197 strongly resemble those of the juvenile *Strobilopterus princetonii* specimen PU 13854 in both armature and ornamentation, the only difference being their comparative increased length in the *Strobilopterus proteus* specimen.

*Strobilopterus proteus* can be differentiated from other species of *Strobilopterus* by the position of the lateral eyes on the outer limits of the central region, compared to their fully central position in *Strobilopterus princetonii* and *Strobilopterus richardsoni* and their centrimesial position in *Strobilopterus laticeps*. The carapace cuticular ornamentation consisting of elongate pustules angling away from the lateral eyes and curving around the carapace margin is clearly present in *Strobilopterus proteus* and *Strobilopterus richardsoni* but appears to be absent from *Strobilopterus princetonii*; the presence or absence of this ornamention cannot be ascertained in *Strobilopterus laticeps* but, given its presence in *Buffalopterus pustulosus*, it is most likely the plesiomorphic condition for the genus. Another difference between *Strobilopterus proteus* and *Strobilopterus princetonii* is that the latter possesses serrations on podomere VI-7a and has a longitudinally drawn-out VI-9, both of which are lacking in *Strobilopterus proteus*.

Infraorder DIPLOPERCULATA Lamsdell, Hoşgör & Selden, 2013

Superfamily PTERYGOTOIDEA Clarke and Ruedemann, 1912

Family PTERYGOTIDAE Clarke and Ruedemann, 1912

Genus *Jaekelopterus* Waterston, 1964

### Type species

*Pterygotus rhenaniae* Jaekel, 1914, by original designation.

### Included species

*Jaekelopterus howelli* (Kjellesvig-Waering and Størmer, 1952b), *Jaekelopterus marylandicus* (Kjellesvig-Waering, 1964).

### Stratigraphical range and distribution

Silurian to Lower Devonian (Wenlock to Emsian) of Maryland and Wyoming, USA, and Alken an der Mosel, Germany.

### Emended diagnosis

Pterygotidae with triangular telson; principal denticles on cheliceral ramus inclined (emended from Waterston [67]).

*Jaekelopterus howelli* (Kjellesvig-Waering and Størmer, 1952)

**Figure 17:**
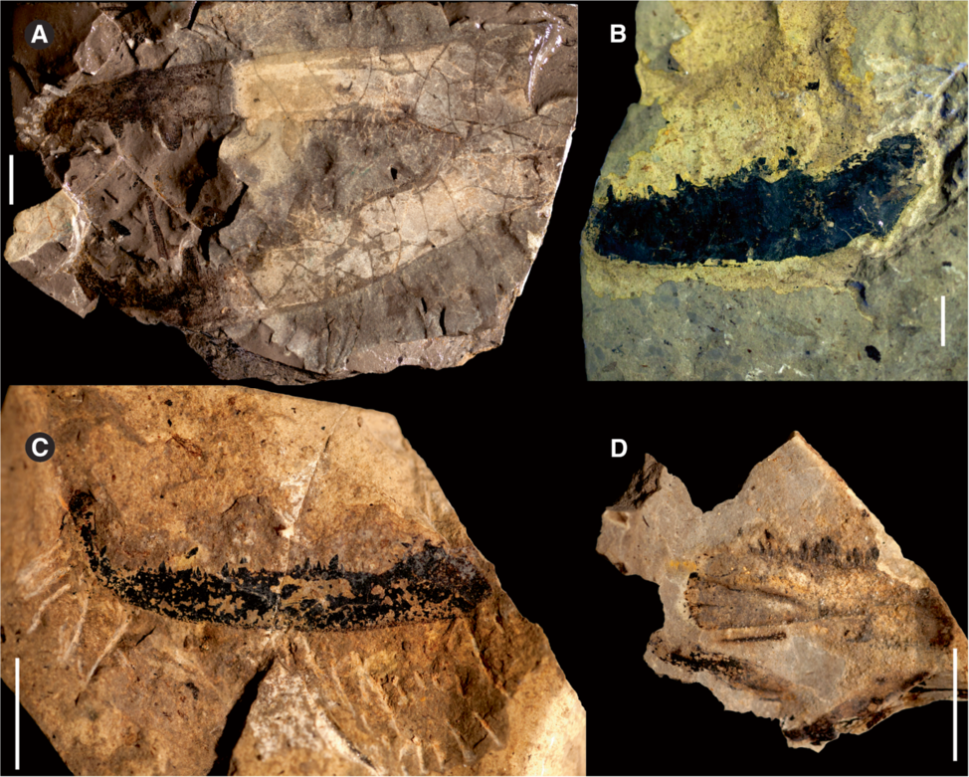
***Jaekelopterus howelli.*** Chelicera specimens. A: FMNH PE 9436. B: KUMIP 292563. C: FMNH PE 26078. D: FMNH PE 61161. Scale bars = 10 mm.

**Figure 18:**
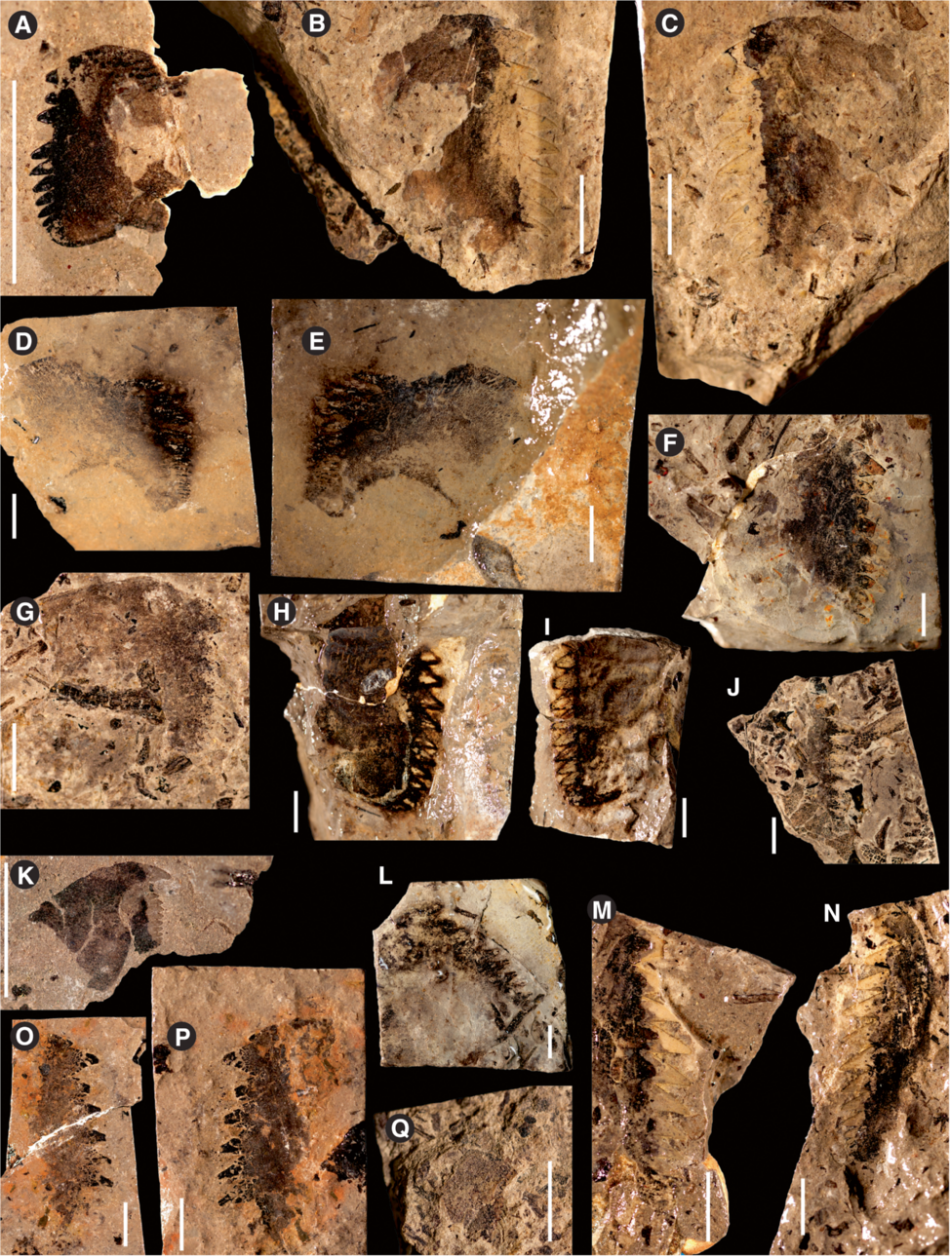
***Jaekelopterus howelli.*** Coxa specimens. **A**: FMNH PE 61183. **B**: FMNH PE 60395. **C**: Counterpart to FMNH PE 60395. **D**: FMNH PE 61181. **E**: Counterpart to FMNH PE 61181. **F**: FMNH PE 9245. **G**: FMNH PE 9238. **H**: FMNH PE 61182. **I**: Counterpart to FMNH PE 61182. **J**: FMNH PE 9241. **K**: FMNH PE 61176. **L**: FMNH PE 9239. **M**: FMNH PE 61186. **N**: Counterpart to FMNH PE 61186. **O**: FMNH PE 61184. **P**: Counterpart to FMNH PE 61184. **Q**: FMNH PE 9240. Scale bars = 10 mm.

**Figure 19:**
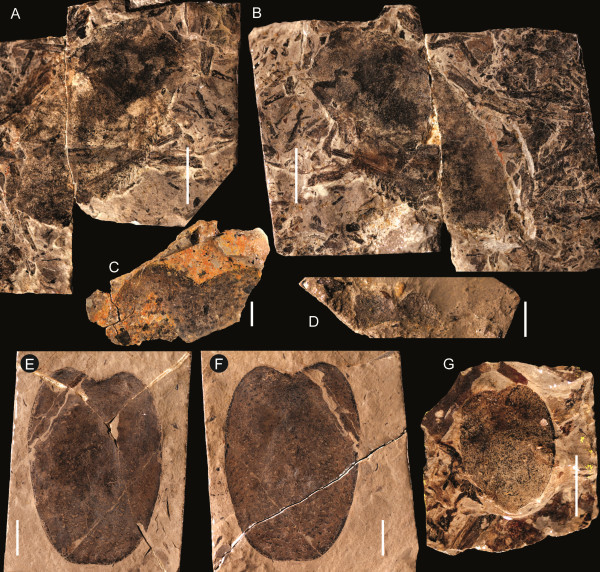
***Jaekelopterus howelli.*** Appendage VI and metastoma specimens. **A**: FMNH PE 61156, paddle. **B**: Counterpart to FMNH PE 61156. **C**: FMNH PE 61169, anterior of metastoma. **D**: FMNH PE 61175, anterior of metastoma. **E**: FMNH PE 61153, metastoma. **F**: Counterpart to FMNH PE 61153. **G**: FMNH PE 61165, metastoma. Scale bars = 10 mm.

**Figure 20:**
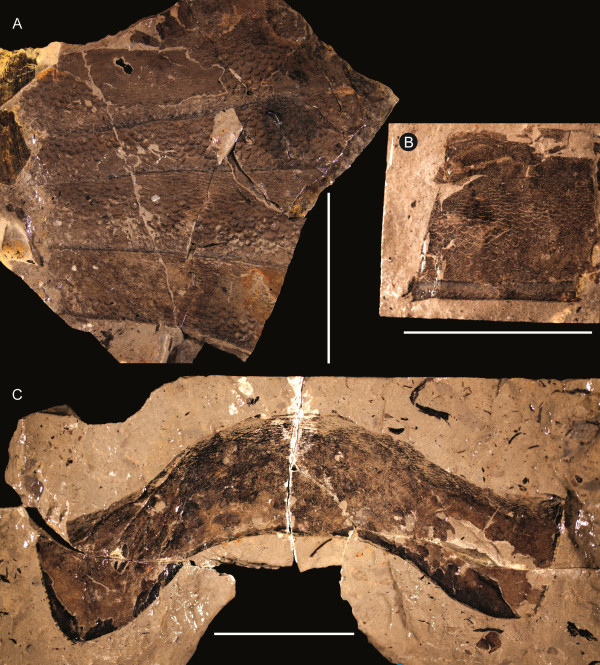
***Jaekelopterus howelli.*** Opisthosomal segment specimens. **A**: FMNH PE 61190. **B**: FMNH PE 7076. **C**: FMNH PE 61189. Scale bars = 50 mm.

**Figure 21:**
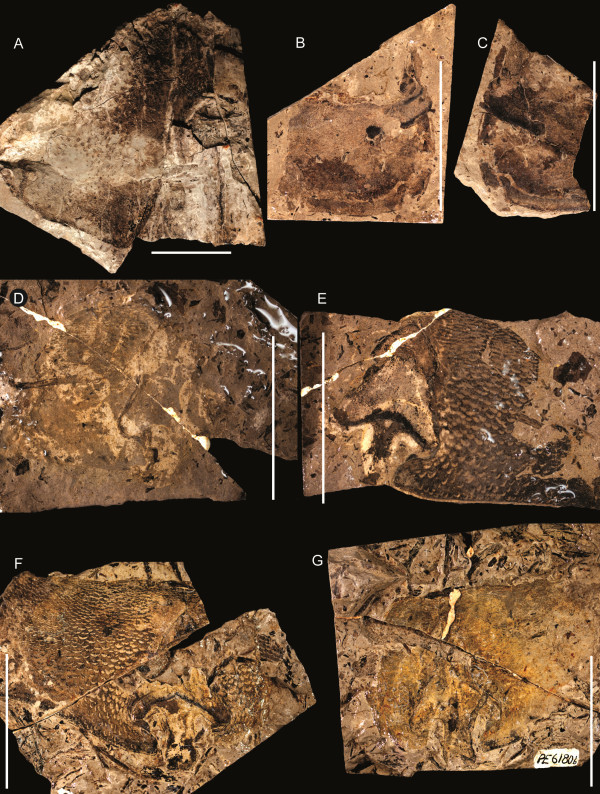
***Jaekelopterus howelli.*** Genital operculum specimens. **A**: FMNH PE 61193, type A operculum. **B**: FMNH PE 61164, tip of type A appendage. **C**: Counterpart to FMNH PE 61164. **D**: PE 6179, type B operculum. **E**: Counterpart to FMNH PE 6179. **F**: FMNH PE 6180, type B operculum. **G**: Counterpart to FMNH PE 6180. Scale bars = 50 mm.

**Figure 22:**
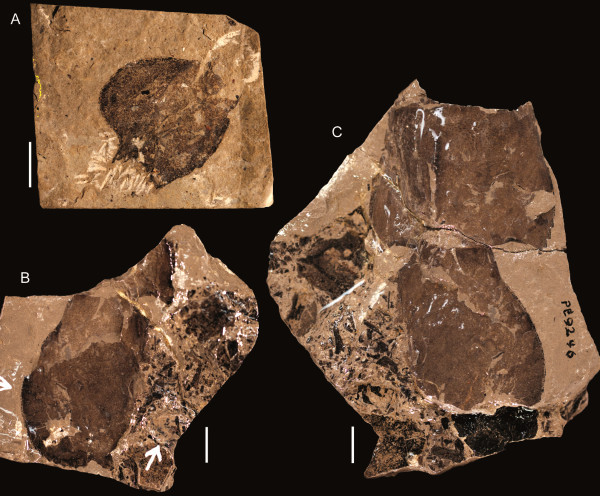
***Jaekelopterus howelli.*** Telson specimens. **A**: FMNH PE 61152. **B**: FMNH PE 9246. **C**: Counterpart to FMNH PE 9246. Scale bars = 10 mm.

Figures 17, 18, 19, 20, 21, 22.

p 1934 *Pterygotus princetonii* Ruedemann, pl. 2 [non pp.163–167, pls. 1 & 3 = *Strobilopterus princetonii* (Ruedemann, 1934)]

* 1952 *Pterygotus (Pterygotus) howelli* Kjellesvig-Waering and Størmer, pp. 997–998, fig. 1

*1964 Pterygotus (Pterygotus) howelli* Kjellesvig-Waering, tables 1 and 2

v. *1986 Pterygotus mcgrewi* Kjellesvig-Waering and Richardson *in* Kjellesvig-Waering, p. 73 [nomen nudum]

*2007 Jaekelopterus* (?) *howelli* Tetlie, p. 1430

v. 2010 *Jaekelopterus* cf. *howelli* Lamsdell and Legg, pp. 1206–1207, fig. 1

### Material

Holotype: YPM 204946 (originally PU 13740), posterior of telson. Additional Material: YPM 204945 (originally PU 13661), FMNH PE 6177.2, PE 6179–6180, PE 7076, PE 9436, PE 9238–9241, PE 9245–9246, PE 26078, PE 60395, PE 61152–61153, PE 61156, PE 61161, PE 61164–61165, PE 61169, PE 61175–61176, PE 61181–61184, PE 61186, PE 61189–61190, PE 61193, KUMIP 292563.

### Horizon and locality

Specimens YPM 204945 and 204946 were collected by Erling Dorf in 1932 from the type section of the Beartooth Butte Formation at Beartooth Butte, Park County, Wyoming, and are Emsian in age. The remaining Field Museum material originates from excavation of the Beartooth Butte Formation section at Cottonwood Canyon, Big Horn County, Wyoming, by Robert H. Denison and Eugene S. Richardson, Jr. in 1962 and is Pragian in age. The University of Kansas specimen is also from the Cottonwood Canyon locality and was collected during fieldwork led by Hans-Peter Schultze in 1983.

### Diagnosis

*Jaekelopterus* with serrated telson margin; second intermediate denticle massively elongate in larger instars; type A genital appendage without median distal indentation.

### Description

*Jaekelopterus howelli* is known in total from 33 specimens, which reveal details of the chelicera, appendage VI, metastoma, genital appendage, opisthosomal tergites, and pretelson and telson. The material from Beartooth Butte is scant, consisting of only the holotype YPM 204946 (the posteriormost portions of a telson) and YPM 204945 (isolated trunk tergite). The Beartooth Butte material is not restudied here; instead, see Kjellesvig-Waering and Størmer [6] for a full description of these specimens, and Ruedemann [19] for a photograph of the holotype. Similarly, the cheliceral ramus referred to *Jaekelopterus* cf. *howelli* (FMNH PE 6177.2) by Lamsdell and Legg [31] is not refigured and reference should be made to that paper for a full account of the specimen. The ramus is, however, herein assigned to *Jaekelopterus howelli* without reservation and measurements of the specimen are presented alongside those of the newly described chelicerae.

No details of the dorsal carapace or visual structures are preserved. Of the ventral prosomal structures only the chelicerae, coxa, distal paddle of appendage VI, and the metastoma are preserved. The chelicerae are represented in five specimens, including the one described by Lamsdell and Legg [31]; four of these are isolated free rami, while one is a fully articulated chelicera consisting of the fixed and free rami (Figure 17). Two of the specimens (FMNH PE 26078 and PE 61161) are from smaller, juvenile individuals (ramus length < 40 mm), while the complete chelicera (FMNH PE 9436), KUMIP 292563 and FMNH PE 6177.2 are from larger, presumably adult, instars (ramus length 90–110 mm) (Table 5). The free ramus is consistent between the juvenile and adult morphologies in possessing a terminal denticle along with three principal and five intermediate denticles. The terminal denticle is oriented almost at a 90° angle to the ramus, while the principal denticles curve posteriorly along their anterior edge so that they are angled away from the terminal denticle. Paired intermediate denticles are located in front of and behind the anterior principal denticle, with a single intermediate denticle at the posterior of the ramus. In the juvenile specimens, the principal and intermediate denticles are more uniform, being of similar length and morphology; however, in the adult specimens, there is strong differentiation between and within the principal and intermediate denticles. The principal denticles are enlarged compared to the intermediate denticles (with the exception of the second intermediate denticle), with the primary denticle being almost twice as broad as either of the other principal denticles. The intermediate denticles are almost invariably half the size of the principal denticles; however, the second intermediate denticle is drastically elongated, being twice the length of any of the principal denticles but retaining the general intermediate denticle width, making it more of a long stiletto in contrast to the broad, slicing blades of the principal denticles or the short teeth of the other intermediate denticles. The only known fixed ramus is from the adult specimen FMNH PE 9436 (Figure 17A), in which the denticle morphology broadly parallels that of the free ramus, with three principal denticles and five intermediate denticles arrayed in the same configuration and being of similar dimensions (Table 6). The fixed ramus differs primarily in the morphology of the terminal denticle, which is angular in comparison to the rounded terminal denticle of the free ramus but retains its 90° angle in relation to the ramus, and in the form of the second intermediate denticle which is not elongated as in the free ramus. The positioning of the denticles on the fixed ramus would result in overlap of the principal denticles when the chelicera was closed, while the intermediate denticles would align but fall short of occlusion.

**Table 5:** ***Jaekelopterus howelli *free ramus measurements**

***Specimen***	***L/W***	***td′***	***d1′***	***d2′***	***d3′***	***i1′***	***i2′***	***i3′***	***i4′***	***i5′***
FMNH	108/24	11*/6*	11/9	10/6	3*/4	2*/2	21/2	5/4	5/4	5/3
PE 9436		–	43	17	62	9	13	30	35	68
FMNH	91/18	10/6	7/5	7/4	2*/3*	2/2	4*/3	2/3	4/4	2/2
PE 6177.2		–	35	14	53	6	9	25	32	69
FMNH	34/8	5/2*	1*/3	3/2	2/1	1/1	1/1	1/1	2/1	1/1
PE 26078		–	14	6	19	3	5	10	12	22
FMNH	24*/7*	−/−	3/3	−/−	3/1	−/−	−/−	2/1*	2/1	−/−
PE 61161		–	–	–	–	–	–	–	–	–
KUMIP	73*/20	−/−	−/−	−/−	−/−	−/−	−/−	−/−	−/−	−/−
292563		–	–	–	–	–	–	–	–	–

**Figure 23:**
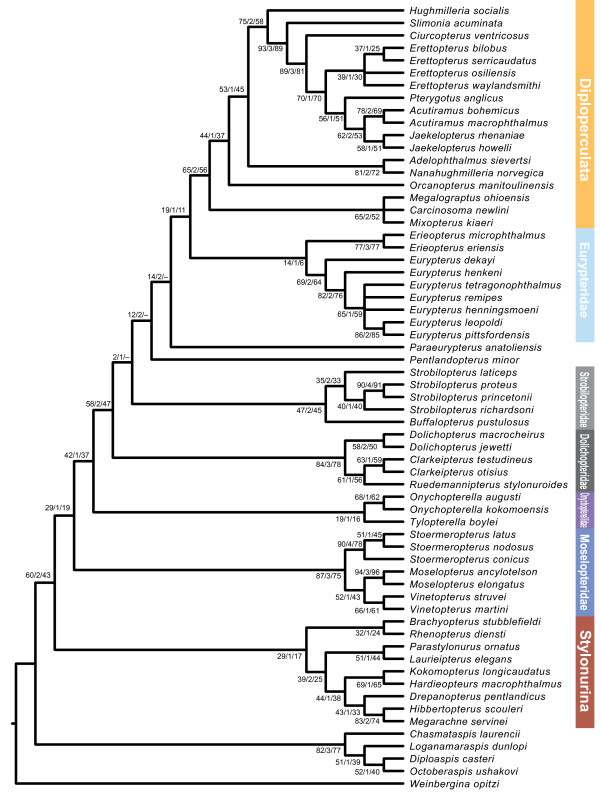
**Result of the phylogenetic analysis.** Strict consensus of 2 MPTs. Clade names are shown to the right of the tree. Diploperculata comprises the clades Mixopteroidea, Adelophthalmoidea and Pterygotoidea. Support values are shown by each node in the following format: jackknife support/bremer support/bootstrap support.

All measurements in millimetres. Column headings correspond to the abbreviations listed in Figure 23. Values in the second row indicate distance from the terminal denticle. Asterisk (*) indicates an incomplete measurement.

**Table 6:** ***Jaekelopterus howelli *fixed ramus measurements**

***Specimen***	***L/W***	***td***	***d1***	***d2***	***d3***	***i1***	***i2***	***i3***	***i4***	***i5***
FMNH	110*/32*	10*/7	10/9	8/6	5/4	5/3	3/2	2*/4	4/4	2*/2
PE 9436		–	46	18	62	8	14	33	38	67

**Figure 24:**
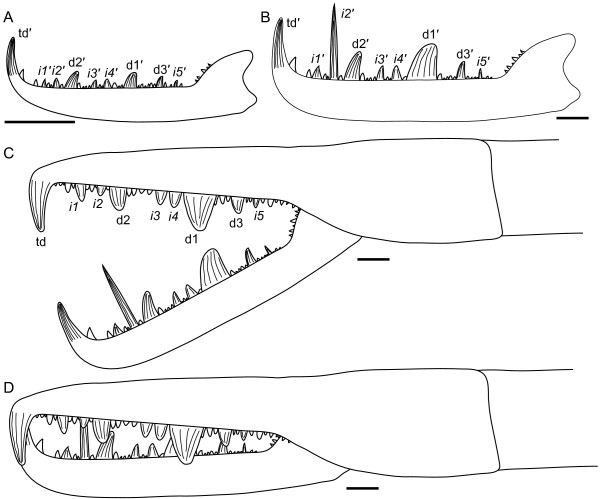
***Jaekelopterus howelli *chelicera.** Reconstruction of the juvenile and adult morphology of the chelicera. **A**: Juvenile free ramus. **B**: Adult free ramus. **C**: Adult articulated fixed and free ramus, agape. **D**: Adult articulated fixed and free ramus, occluded. Abbreviations: td = terminal denticle of fixed ramus; d1–d3 = principal denticles of the fixed ramus; i1–i5 = intermediate denticles of the fixed ramus; td′ = terminal denticle of the free ramus; d1′–d3′ = principal denticles of the free ramus; i1′–i5′ = intermediate denticles of the free ramus. Scale bars = 10 mm.

All measurements in millimetres. Column headings correspond to the abbreviations listed in Figure 24. Values in the second row indicate distance from the terminal denticle. Asterisk (*) indicates an incomplete measurement.

The postoral prosomal appendages are known only from a single coxa of appendage IV or V and a number of fragmentary specimens of appendage VI (Figure 18). The coxa of IV/V (FMNH PE 61181) has a preserved length of 44 mm, with a width of 28 mm at the gnathobase and a preserved width of 29 mm distally. Twenty teeth are preserved at the gnathobase; these have a uniform long, narrow morphology and are somewhat curved. The coxa narrows markedly after the gnathobases before expanding distally. Eleven coxae of appendage VI are preserved, ranging in length from 8–50 mm (Table 7), representing both juvenile and more mature individuals. The morphology of coxa VI differs from that of coxa IV/V in being comparatively broader and having larger, more robust teeth, the most anterior of which is enlarged compared to the succeeding teeth. There are also fewer teeth constituting the gnathobase; the most complete large coxa (FMNH PE 61186) preserves 13 teeth while some of the smaller specimens possess only 10–11 teeth, suggesting that teeth continued to develop later in ontogeny. The smallest coxa (FMNH PE 61176) also differs in the morphology of the teeth, which lack the curved anterior margin seen in larger specimens, while the largest coxae show signs of an ancillary tooth forming alongside the anterior enlarged tooth. Distally, appendage VI is only known from a single specimen (FMNH PE 61156) that preserves the two distal podomeres of a swimming paddle (Figure 19A, B). The specimen has a preserved length of 51 mm and a maximum width of 25 mm and consists of podomere VI-8 with podomere VI-9 roughly preserved. The anterior margin of VI-8 bears uniform, distally angled serrations while VI-9 is set into a notch located towards the posterior side of the distal margin of VI-8.

**Table 7:** ***Jaekelopterus howelli *coxa measurements**

***Specimen***	***Length***	***Width (gnathobases)***	***Width (distally)***
FMNH PE 9238	32*	20*	30*
FMNH PE 9239	50*	47*	47*
FMNH PE 9240	9*	10*	11*
FMNH PE 9241	24*	37*	11*
FMNH PE 9245	25*	36*	39*
FMNH PE 60395	30*	30*	32*
FMNH PE 61176	9*	6	7*
FMNH PE 61182	37*	43*	52*
FMNH PE 61183	8*	9	9*
FMNH PE 61184	26*	40*	43*
FMNH PE 61186	17*	39*	48*

All measurements in millimetres. Asterisk (*) indicates an incomplete measurement.

The metastoma is represented by four specimens (Table 8), two of which (FMNH PE 61169 and PE 61175) only preserve the anterior portion (Figure 19). The two complete specimens are oval with their widest point being located at the centre, a rounded posterior margin, and shallow anterior notch flanked by rounded shoulders. The notch in all specimens has a median angle of 120–135°, with the exception of FMNH PE 61175 (Figure 19D), which is laterally compressed. Of the two complete specimens, FMNH PE 61153 (Figure 19E) is large, with a length of 57 mm, and FMNH PE 61175 would likely have been of a similar size when complete; however, FMNH PE 61169 (Figure 19C) would easily have been twice as large. The second complete metastoma, FMNH PE 61165 (Figure 19G), is smaller (length 20 mm), however, and differs from FMNH PE 61153 (Figure 19E, F) in being comparatively broader, having a length/width ratio of 1.42 compared to the ratio of 1.46 in FMNH PE 61153.

**Table 8:** ***Jaekelopterus howelli *metastoma measurements**

***Specimen***	***Length***	***Width (centre)***	***Width (base)***	***Width (shoulders)***	***Notch depth***	***Notch angle***
FMNH PE 61153	57	39	16	17	4	130º
FMNH PE 61165	20	14	4	6	1	135º
FMNH PE 61169	45*	60*	–	36	10	120º
FMNH PE 61175	10*	23*	–	10*	5	80º*

All measurements in millimetres. Asterisk (*) indicates an incomplete measurement.

Three specimens preserve details of the opisthosomal tergites (Figure 20), all identifiable from the typical pterygotid ornamentation of large scales that grade from being broad or chevron-shaped anteriorly to more elongate semilunate and linguoid scales posteriorly that is also seen on the isolated tergite from Beartooth Butte (YPM 204945). One specimen (FMNH PE 7076) is simply a fragment of cuticle, 40 mm long and 63 mm wide; however, FMNH PE 61189 (Figure 20C) is a complete tergite and FMNH PE 61190 (Figure 20A) consists of a number of tergites in series with their lateral margins missing. FMNH PE 61190 is 218 mm long in total, with a maximum preserved width of 211 mm, and preserves four tergites with the following length/width measurements (asterisks indicate incomplete measurements): 47 mm/200 mm*, 40 mm/211 mm*, 54 mm/162 mm*, 47 mm/133 mm*. The first tergite shows the smooth articulating facet across its anterior border with a row of flattened scales delineating the posterior extent of the articulation, while all the tergites also display a cuticular thickening at the posterior margin that can also be seen in FMNH PE 7076 (Figure 20B). The isolated tergite FMNH PE 61189 has a length of 49 mm and a width of 181 mm and shows strong curvature between the axial and pleural regions, with the lateral margins of the tergite appearing swept back, probably a genuine characteristic in life magnified by the flattening of its three-dimensional shape during the taphonomic process. The lateral margins of the tergite are ornamented with a row of rectangular scales that give it a crenate (*sensu* Tollerton [42]) outline.

The only ventral opisthosomal structures preserved are the genital operculum and genital appendage, with material of both the type A and type B morphologies present (Figure 21). The type A material is known from two specimens, both of which are fragmentary and relatively poorly preserved. FMNH PE 61193 (Figure 21A) consists of the medial portions of a genital appendage and one ala (*sensu* Wills [68]) and has a preserved length of 144 mm with a preserved width of 158 mm. The ala is broad, curving smoothly away from the genital appendage distally, and is a single plate lacking the suture that marks the median and posterior opercular plates in some taxa. The lateral and distal margins of the ala show a thickening of the cuticle which narrows towards the base of the genital appendage. The anterior portions of the operculum are not preserved, so it is impossible to see whether deltoid plates were present. The ornamentation of the operculum consists of lunate scales that angle distally while following the curvature of the ala margin away from the genital appendage. The genital appendage itself is long and narrow, having a preserved length of 134 mm and a proximal width of 23 mm, and appears to consist of a single ventral lamella lacking segmentation which extends beyond the posterior margin of the operculum, thickening distally (distal width 33 mm). The other type A genital appendage, FMNH PE 61164 (Figure 21B, C), is the tip of the ventral lamella of an exceptionally large individual, with a width of 46 mm and a preserved length of 54 mm. The lamella is bordered by a doublure distally that is 5 mm thick and narrows as it curves up the lateral margins of the lamella. The distal termination of the lamella is rounded with no evidence of bilobation; however, the lamella clearly begins to narrow anteriorly, indicating it possessed the typical pterygotid spoon-shape. Another structure possessing doublure or cuticle thickening overlies the lamella partway up its preserved length. This structure is better preserved in the counterpart which shows the cuticle to be ornamented with scales and suggests that the structure is in fact a displaced ala from the genital operculum.

The type B genital appendage is also known from two specimens, both of which preserve a single ala of the genital operculum and the genital appendage itself. Both specimens are large, ranging from 81–175 mm in preserved width with a maximum length of 54–79 mm (Table 9). The ornamentation of the operculum consists of broad scales that become more elongate and linguoid towards the segment posterior; there is no differentiation between median and posterior opercular plates, the operculum appearing to be a single plate lacking any joining suture as in the type A individuals. A cuticular thickening or doublure is present around the distal and inner margin of the operculum, especially prominent where the ala is in contact with the genital appendage. The genital operculum also curves distally around the genital appendage, encompassing the broader upper part of the appendage and abutting the distal lamella. The counterpart specimen of FMNH PE 6179 (Figure 21E) shows clear indication of triangular deltoid plates flanking the anterior portion of the genital appendage, although the sutures are not clear on the part; the other specimen, FMNH PE 6180 (Figure 21F) also displays deltoid plates but the sutures are only faintly preserved and identifying the plates is made harder as there is no break or differentiation in cuticular ornamentation. The type B genital appendage at first glance appears to be composed of two units, a proximal dorsal segment and an underlying lamella that comprises the main portion of the appendage; however, closer study reveals that these are, in fact, internal structures, and that the true ventral lamella has broken off near its base in both specimens. The ventral lamella is most obvious in FMNH PE 6179 (Figure 21D) where its remnants are preserved at the anterior of the appendage, preserving a clear suture where it attached to the operculum with the lamella cuticle being the same colour and bearing similar ornamentation as that of the adjoining ala. The lamellar cuticle is then broken away to reveal the internal structures of the appendage, which are preserved at a different level to the surrounding operculum. The most prominent internal structure, the anterior portion with a broad flange-like termination marked by a distinct thickening, represents the internal doublure or cuticular folding of the operculum which is positioned dorsal to the genital appendage but has been superimposed onto the ventral structures due to compression. The other structure represents the dorsal plate of the genital appendage and consists of a broad triangular plate that narrows drastically posterior to the operculum internal doublure before lengthening distally into a spoon-shaped extension with a bilobed termination. The shape of the true ventral lamella (and hence the genital appendage) is, therefore, uncertain; however, FMNH PE 6179 clearly preserves a hastate proximal portion. The shape of the dorsal plate is a reliable proxy for the distal shape of the ventral lamella, however cuticular fragments around the spoon-shaped portion of the plate in FMNH PE 6180 suggests the ventral lamella did not narrow as suddenly as the dorsal plate does, instead filling the available space between the two ala of the operculum and forming an uneven diamond.

**Table 9:** ***Jaekelopterus howelli *type B genital operculum measurements**

***Specimen***	***Length (centre)***	***Length (lateral)***	***Width***	***Appendage length***	***Appendage width (centre)***	***Appendage width (distal)***
FMNH PE 6179	54*	–	81*	48	32	11
FMNH PE 6180	79	57	175*	71	45	14

All measurements in millimetres. Asterisk (*) indicates an incomplete measurement.

The telson of *Jaekelopterus howelli* is known from two specimens at Cottonwood Canyon which correspond well to the specimen from Beartooth Butte (Figure 22). FMNH PE 9246 consists of an articulated telson and pretelson with a total preserved length of 89 mm, the pretelson being 37 mm long and 46 mm wide and the telson 52 mm long with a preserved width of 26 mm. The pretelson is shown to have serrated lateral margins and bears a median carina, while the telson broadens distally and has a flattened posterior margin, hinting at a more triangular shape. The lateral and posterior margin of the telson also bears serrations formed by angular scales. The presence or otherwise of a median carina cannot be ascertained; however, a flattened, ridge-like structure that runs down the centre of the telson may be this feature, although it could also be the result of taphonomic cuticular folding. The second Cottonwood Canyon specimen (FMNH PE 61152) is a relatively complete, isolated telson with a total length of 30 mm (24 mm discounting the terminal spine) and a width of 11 mm at its base, expanding to 23 mm distally. The posterior margin of the telson is almost flat, giving it a definite triangular outline. The lateral and posterior margins are ornamented with angular scales resulting in a serrate margin; these scales reduce in size towards the median posterior spine, which is triangular and 6 mm long, being 4 mm wide at its base. The dorsal surface of the specimen is somewhat worn, and although a structure resembling a median carina is present, it is far from definitive. This specimen, in particular, closely resembles the holotype Beartooth Butte telson (YPM 204946), which is far larger then either of the specimens described here, being 128 mm in width. The holotype also possesses a flattened posterior margin with serrated margins caused by the presence of angular scales that decrease in size towards a triangular posterior spine.

### Remarks

*Jaekelopterus howelli* shares an almost identical cheliceral denticulation pattern with *Jaekelopterus rhenaniae* along with a flattened posterior margin to the telson, resulting in an overall triangular shape. The Cottonwood Canyon species is clearly differentiated, however, through its possession of a serrated margin to the telson and the massive elongation of the second intermediate denticle in larger instars. The juvenile chelicerae clearly exhibit the *Jaekelopterus* denticulation pattern and show similar trends to those noted in juvenile chelicerae of *Jaekelopterus rhenaniae* by Poschmann and Tetlie [69]: specifically, a more gracile terminal denticle and less differentiation between the principal and intermediate denticles. The form of the FMNH PE 61152 telson (Figure 22A), with its flattened posterior, triangular outline and serrated margins, confirms that the Cottonwood Canyon species is the same as the Beartooth Butte pterygotid.

### Phylogenetic affinities

Analysis of the phylogenetic matrix as detailed in the methods section yielded two most parsimonious trees with a tree length of 314 steps, an ensemble Consistency Index of 0.455, ensemble Retention Index of 0.811, and Rescaled Consistency Index of 0.369, the strict consensus of which is presented here (Figure 23). The topology is predominantly congruent with that retrieved by Lamsdell *et al.*[47], while the intrarelationships of the expanded Stylonurina is the same as in earlier analyses [48,49] and the resolution of the pterygotoids is identical to the analysis of Braddy *et al.*[51]. The result differs from previous hypotheses in splitting the two constituent clades of Dolichopteridae, resulting in the family as presently defined being paraphyletic. The more basal clade consists of *Dolichopterus* Hall, 1859, *Ruedemannipterus* Kjellesvig-Waering, 1966 and *Clarkeipterus* Kjellesvig-Waering, 1966 and comprises the pruned Dolichopteridae, defined by the possession of antelaterally (*sensu* Tollerton [42]) positioned lateral eyes, an angle between podomeres VI-3 and VI-4 other than 180°, an angular projection on the anterior of VI-7, an additional moveable lobe on VI-8, and an expanded VI-9. The second clade constitutes the newly named Strobilopteridae and includes *Strobilopterus* and *Buffalopterus*. The clade is defined by the possession of a semicircular carapace, the first podomere of appendage VI that fully projects beyond the carapace margin being VI-6 (as opposed to VI-4 as in most eurypterids), a distinctive carapace ornament consisting of elongate scales that angle away from the lateral eyes, and an ornamentation of angular scales across the posterior of the tergites. Strobilopterids are a node closer to Diploperculata in relation to dolichopterids due to podomere VI-7a being more than half the width of VI-7 and VI-9 being less than 25% the length of VI-8 (although this characteristic is reversed in adult *Strobilopterus proteus* and *Strobilopterus princetonii*, it is present in earlier ontogenetic stages of *Strobilopterus princetonii*[5]).

All three taxa that are included in the phylogeny for the first time resolve within established clades. *Jaekelopterus howelli* is the sister-taxon to *Jaekelopterus rhenaniae*, united by the possession of a triangular-shaped laterally expanded telson. *Strobilopterus proteus* is united with *Strobilopterus princetonii* by a suite of characters including the presence of carapace genal facets, a large podomere VI-9 which is greater than a quarter of the length of podomere VI-8, podomere VI-9 bearing serrations, and possibly the presence of an additional moveable lobe on VI-8. This last character is uncertain, however, as it is possible that the lobe in these taxa is a fixed extension of the podomere lacking the articulation reported in *Dolichopterus*. *Strobilopterus laticeps*, on the other hand, resolves at the base of the *Strobilopterus* clade, united by the presence of angular spatulae associated with the type A genital appendage and the occurrence of broad, sclerotised lunate scales in congruence with the striate opercular ornamentation. The position of *Strobilopterus laticeps* at the base of the clade is important because it suggests that characters that previously grouped strobilopterids with dolichopterids, such as an enlarged podomere VI-9 and serrated podomeres on limb VI, are not part of the *Strobilopterus* groundplan, and developed convergently in those species that possess them.

## Discussion

### Ontogeny

Chelicerates, like all arthropods, mature through a series of static stages called instars punctuated by periods of ecdysis followed by immediate rapid growth. Unlike many crustaceans and insects, however, chelicerates are generally considered to be direct developers that do not undergo extreme metamorphosis after hatching, although some pycnogonids gain body segments with associated limbs during postembryonic development [70] while extant xiphosurans hatch without their full compliment of opisthosomal appendages [71]. This form of hemianamorphic direct development may be the plesiomorphic condition for euarthropods; it is also observed in basal crustaceans, basal myriapods, and trilobites [72], and may be present in megacheirans [73]; the hexapodal larval stage of Acari and Ricinulei is, however, likely to be an independently derived condition [74]. True direct development was therefore thought to be a characteristic of arachnids; however, the veracity of larval eurypterids apparently showing a reduced segment count [75] is uncertain [76], and so it is unclear whether true direct development had already been attained by eurypterids.

There have been few studies of ontogeny in eurypterids, the most widely cited being that of Andrews *et al.*[7], which focused on *Eurypterus remipes* DeKay, 1825; Brower and Veinus [77] and Cuggy [8] also conducted studies on *Eurypterus remipes*, with similar work also having been conducted on *Hardieopterus* (?) *myops* (Clarke, 1907) by Brower and Veinus [76] and *Adelophthalmus luceroensis* Kues and Kietzke, 1981 [9]. The lack of work focusing on eurypterid ontogeny beyond these few examples probably stems from an apparent lack of juveniles in the fossil record. The co-occurrence of different instars at the Cottonwood Canyon locality therefore appears to be a rarity; aside from *Eurypterus remipes*, *Hardieopterus* (?) *myops*, *Adelophthalmus luceroensis*, and the newly described species herein, only juveniles of *Hughmilleria shawangunk* Clarke, 1907 [75] and *Drepanopterus pentlandicus* Laurie, 1892 [50] have also been reported. However, the influence of ontogeny has rarely been considered among chelicerate palaeontologists when describing species as has been shown recently in the case of the xiphosurid genus *Euproops* Meek, 1867 [10], and there remains the strong possibility that a number of eurypterid species are oversplit taxonomically. It has already been suggested that *Pterygotus minor* Woodward, 1864 is a juvenile of *Pterygotus anglicus* Agassiz, 1844 (the two species were synonymised by Braddy [78]), and with *Erieopterus brewsteri* Woodward, 1864 also received the same treatment as a juvenile specimen of *Tarsopterella scotica* (Woodward, 1872). Further probable synonyms remain: *Eusacarna obesa* (Woodward, 1868) is almost certainly a juvenile form of ‘*Carcinosoma*’ *scorpioides* (Woodward, 1868) from the same locality, while *Moselopterus elongatus* Størmer, 1974 and *Parahughmilleria major* Størmer, 1973 are likely adults of the co-occurring *Moselopterus ancylotelson* Størmer, 1974 and *Parahughmilleria hefteri* Størmer, 1973 respectively (JCL personal observations). *Stylonurella* (?) *arnoldi* (Ehlers, 1935) also exhibits signs of representing a juvenile morphology, including enlarged lateral eyes. No adult eurypterids are found in immediate association, although the large hardieopterid *Hallipterus excelsior* (Hall, 1884) is known from the same formation and may yet prove to be conspecific, although it is known only from its carapace and any suggested affinities are extremely tentative. Finally, a eurypterid from Siberia recently described as *Stylonuroides orientalis* Shpinev, 2012 appears to exhibit genal spines and large lateral eyes while having a carapace breadth of less than 10 mm; given the juvenile material described here, it seems certain that ‘*Stylonuroides*’ *orientalis* is an early juvenile form, possibly of one of the other eurypterids present in the fauna.

It is clear that there is still much work needed in order to tease apart the ontogenetic pathways exhibited by eurypterids; by fully describing the ontogenetic changes occurring in species where it can be observed it is possible to propose general trends that will aid in the identification of juveniles in other assemblages, along with providing support for or against homology statements of various morphological features between different taxa. Within this framework, the Cottonwood Canyon species provide a unique and critical insight into eurypterid ontogeny, with multiple instars and multiple specimens of each instar preserved in species that are phylogenetically removed from the well-studied *Eurypterus*.

### Ontogeny of *Jaekelopterus howelli*

The fragmentary and incomplete nature of the currently available *Jaekelopterus howelli* material makes it impossible to describe the ontogeny of the species in detail, even though more than one instar is present in the assemblage. Nevertheless, observations are possible on changes in the chelicerae, metastoma and telson. The chelicerae are known from five specimens, four showing the denticles of the free ramus in some detail, of which two are interpreted as adults and two as juveniles. While both juvenile and adult morphologies clearly correlate well with each other, possessing the same number of denticles in the same arrangement, there are also a number of marked differences (Figure 24). The principal denticles exhibit positive allometry in relation to the intermediate denticles, being two to three and a half times the size of the intermediate denticles in adult specimens in contrast to juveniles, the principal denticles of which are only one and a half times the size of the intermediate denticles. The terminal denticle shows further positive allometry in comparison to the principal denticles, being larger and more robust in the adult specimens; however, the most extreme example of positive allometry occurs in the second intermediate denticle. This denticle is in no way differentiated from the other intermediate denticles in juvenile specimens, yet in adults it is massively elongate, becoming over twice the length of any of the principal denticles. Positive allometry of the denticles in pterygotid chelicerae has been noted before [79]; however, there has been no published in-depth study on the development of the chelicerae through ontogeny. At present, the extreme elongation of the second intermediate denticle appears unique among eurypterids, although positive allometry of the terminal and principal denticles may be commonplace among pterygotids.

The metastoma also appears to alter its dimensions throughout ontogeny. The juvenile metastoma appears comparatively broader than that of the adult, yet the length/width ratios are not drastically different, being 1.43 as opposed to 1.46. A decrease in relative width of the metastoma through ontogeny has, however, been shown in the related species *Jaekelopterus rhenaniae*[69], and the presence of larger, incomplete metastomae at Cottonwood Canyon means that its final length/width ratios may have been higher still. There may be another change occurring, with the angle of the anterior notch becoming more acute in large specimens, decreasing from 135° in the juvenile metastoma to 120° in the largest specimen. This may, however, simply be another expression of the metastoma getting comparatively narrower through ontogeny, as the angle is defined by the breadth of the flanking shoulders. As the metastomal width decreases, so does that of the shoulders, which makes the notch angle more acute. A decrease in the relative width of the metastoma through ontogeny has also been noted in *Stoermeropterus* Lamsdell, 2011 and *Moselopterus* Størmer, 1974 [64] and, while it appears that the metastoma in *Eurypterus* retains its dimensions throughout ontogeny [80], the new evidence here from *Jaekelopterus* and *Strobilopterus* (below) suggests that the stasis in *Eurypterus* may be the exception rather than the rule.

### Ontogeny of *Strobilopterus proteus*

**Figure 25:**
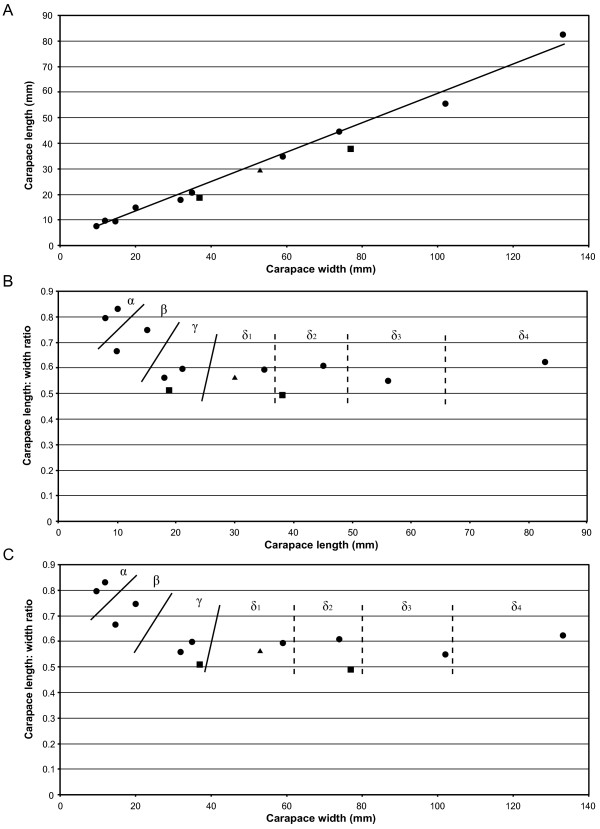
**Graphs comparing carapace dimensions of instars of *Strobilopterus.*** Circles represent specimens of *Strobilopterus proteus*, while triangles represent *Strobilopterus richardsoni* and squares represent *Strobilopterus princetonii*. **A**: Carapace length vs. carapace width. The regression line is length = 0.5825(width) + 1.4404 and is statistically significant (r^2^ = 0.99034, 8 degrees of freedom, p = 2.39×10^-9^) indicating that the relationship between carapace length and carapace width is not random and therefore likely due to ontogeny. **B**: Carapace length:width ratio vs. carapace length. Instar groupings are shown, with the potential subdivision of the δ specimens shown with dashed lines. **C**: Carapace length:width ratio vs. carapace width. Instar groupings are shown, with the potential subdivision of the δ specimens shown with dashed lines.

*Strobilopterus proteus* offers a more complete, although still far from comprehensive, record of post-embryonic development in a eurypterid species. The *Strobilopterus* material at Cottonwood Canyon comprises at least 13 individuals, as derived from a simple carapace count, which encompass a broad range of sizes. Recognising moult stages in eurypterids can be difficult, as like in xiphosurans they are not always clearly discontinuous; however, it has been shown that it is possible to separate instars based on carapace dimensions [7]. In an attempt to differentiate instars of *Strobilopterus proteus*, measurements of carapace length were compared to carapace width (Figure 25A). The resulting scatterplot suggests a number of groupings that may represent instars, although the smallest individuals fall very close together and are difficult to separate. What the plot does indicate, however, is that the carapace dimensions fall fairly neatly along the regression line, suggesting that any difference in relative dimensions is more likely to be due to an ontogenetic trend rather than taphonomic distortion which produces a more random distribution.

In order to further distinguish the possible instar groupings, comparisons of carapace length and width to the carapace length/width ratio were carried out (Figure 25B, C). These both revealed the same sets of groupings which are interpreted here as being true instars; a possible larval stage (termed α), a juvenile stage (β), a later juvenile or subadult stage (γ) and a final subadult to adult stage (δ). This final stage can potentially be broken down into a further four stages (δ_1_– δ_4_), all of which maintain the same adult morphology; however, as each potential instar is represented by only a single *Strobilopterus proteus* specimen, their identification at this time is extremely tentative. If these do represent instars, then this likely indicates that *Strobilopterus* attained its full adult morphology before sexual maturity, as neither modern horseshoe crabs [81] nor scorpions [82] moult again once becoming able to reproduce.

Specimens of two other *Strobilopterus* species were also plotted alongside the *Strobilopterus proteus* distribution in order to test if they easily resolve within any of the recognised instars (Table 10). Three specimens were able to be included, each of which fit within a hypothesised moult stage: the holotype of *Strobilopterus richardsoni*, FMNH PE 5120, correlates to the possible δ_1_ stage while *Strobilopterus princetonii* specimen YPM 204949 falls within stage δ_2_. The juvenile specimen of *Strobilopterus princetonii* recognised by Tetlie [5] (PU 13854) resolves within stage γ. While each species probably has a somewhat different ontogenetic trajectory, it has been shown that the disparity in three different species of *Adelopthalmus* is not great [9] and so the instars of *Strobilopterus proteus* are considered a good proxy for those of the other *Strobilopterus* species. Furthermore, the juvenile *Strobilopterus princetonii* corresponds in morphology to the specimens of *Strobilopterus proteus* assigned to stage γ with which it is associated. Therefore, these specimens may be useful in corroborating ontogenetic trends observed in the *Strobilopterus proteus* material.

**Table 10:** Carapace data used in instar analysis

***Specimen***	***Length***	***Width***	***L/W ratio***	***Instar***
FMNH PE 6165	8	*10*	0.80	α
FMNH PE 6166	18	*32*	0.56	γ
FMNH PE 9236	10	15	0.67	β
FMNH PE 28961	83	133	0.62	δ_4_
FMNH PE 61151	56	*102*	0.55	δ_3_
FMNH PE 61154	45	*74*	0.61	δ_2_
FMNH PE 61162	35	*59*	0.59	δ_1_
FMNH PE 61166	21	*35*	0.60	γ
FMNH PE 61197	10	12	0.83	α
FMNH PE 61198	15	*20*	0.75	β
YPM 204949	38	77	0.49	δ_2_
PU 13854	19	37	0.51	γ
FMNH PE 5120	30	53	0.57	δ_1_

Italics represent a carapace width retrieved from extrapolating from half a complete carapace. YPM 204949 and PU 13854 are specimens of *Strobilopterus princetonii*, and FMNH PE 5120 is the holotype of *Strobilopterus richardsoni*. All measurements in millimetres.

**Figure 26:**
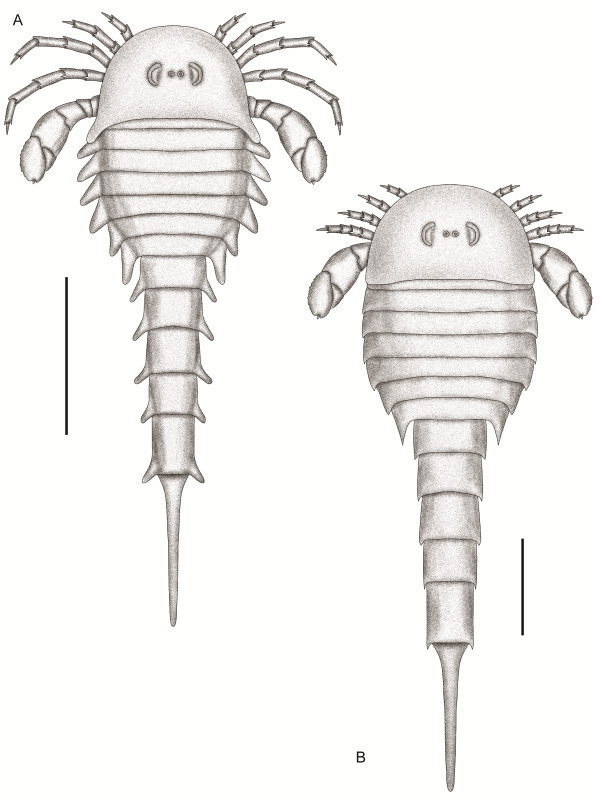
***Strobilopterus proteus *juvenile instars.** Reconstructions of juvenile instars. **A**: Possible larval instar α. Distal morphology of the paddle is extrapolated from a juvenile individual of *Strobilopterus princetonii*. The telson is reconstructed from FMNH PE 61166. **B**: Juvenile instar β. Details of distal paddle morphology are extrapolated from a juvenile individual of *Strobilopterus princetonii*. The telson is extrapolated from FMNH PE 61166. Scale bars = 10 mm.

**Figure 27:**
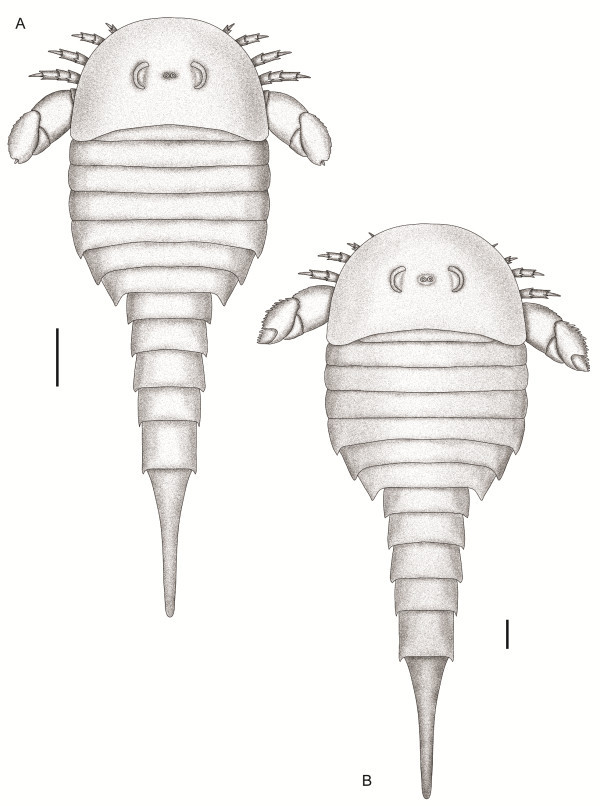
***Strobilopterus proteus *subadult and adult instars.** Reconstructions of subadult and adult instars. **A**: Juvenile/subadult instar γ. Limbs and posterior opisthosomal segments are extrapolated from a juvenile individual of *Strobilopterus princetonii*. The telson is extrapolated from FMNH PE 61166. **B**: Subadult/adult instar δ. Anterior prosomal limbs are extrapolated from *Strobilopterus princetonii*. The telson is extrapolated from FMNH PE 61166. Scale bars = 10 mm.

Using the available instars, it is possible to identify a number of trends operating during the postembryonic development of *Strobilopterus proteus*. The most striking, when comparing the α material to the δ specimens, is the presence of large epimera in the former (Figure 26A). The presence of epimera in these specimens is somewhat inconsistent, with different specimens preserving epimera on the mesosoma, metasoma, or both. It has been noted in other eurypterid species, however, that the lateral epimera tend to break off during collection when the rock is split due to them being positioned on a slightly different plane in the sediment to the main body fossil [83], and this is likely also the case here. The epimera are much reduced in the stage β individuals but are still present as small projections on each of the opisthosomal segments (Figure 26B), while the available γ material shows the epimera to have been completely reduced on at least the second tergite (Figure 27A). By stage δ, all the epimera on opisthosomal segments 2–4 are wholly reduced, with the epimera on segments 5–12 short and mostly vestigial, with the exception of those of the seventh segment which are retained as relatively large, angular projections (Figure 27B). Evidence from the juvenile material of *Strobilopterus princetonii* that can be assigned to stage γ suggests that segments 2–4 are already devoid of epimera but that the epimera on the remaining segments are more pronounced than those seen in δ [5]. The observation that epimera may reduce in size throughout ontogeny has also been reported in the stylonurine eurypterid *Drepanopterus pentlandicus*[50], and a reported juvenile specimen of *Hardieopterus* (?) *myops* figured by Clarke and Ruedemann [75] (their pl. 51, fig. 6) exhibits long epimera on the opisthosomal segments that are apparently absent from the adult specimens. A distinct phenomenon has been noted in the ontogenetic development of the xiphosuran *Euproops*, in which the juveniles possess long epimera, the bases of which expand dorsoventrally in each instar so as to increase the apparent width of the opisthosoma and reduce the size of the epimeral projections [10]. A similar situation appears to occur in *Strobilopterus proteus*, with each instar getting comparatively broader as the epimera decrease in size, and may be characteristic of the chelicerate ground plan.

The other immediately obvious trend is the relative reduction in length of the prosomal appendages as the animal matured. The prosomal appendages of the α specimens are long and gracile, with elongate podomeres that result in the appendages projecting from beneath the carapace margin more proximally than in adult specimens, at around the fourth podomere; this results in appendage V curving back as far as the fifth opisthosomal segment. The paddle of appendage VI also possesses comparatively longer podomeres, also appearing to project from under the carapace at the fourth podomere, as is usual for Eurypterida. The appendages have begun to shorten in the β individuals, with appendage IV projecting from beneath the carapace margin at the fifth podomere while appendage VI emerges at the sixth podomere. None of the known *Strobilopterus proteus* γ stage specimens preserve the opisthosomal appendages; however, the apparent γ *Strobilopterus princetonii* shows both appendage IV and VI appearing at the margin of the sixth and seventh podomeres [5]. In stage δ appendage VI has shortened further still, with the paddle projecting from beneath the carapace at the seventh podomere. The degree of relative shortening of appendage length is extreme in *Strobilopterus proteus*; however, the same general trend has been noted in *Eurypterus remipes*[7] and *Drepanopterus pentlandicus*[50], and can also be observed in the material assignable to ‘*Carcinosoma*’ *scorpioides*[84]. A similar trend can also be seen in the modern xiphosuran *Limulus*, the early free-swimming instars of which have comparatively longer prosomal appendages than the benthically inclined adults [81]. *Strobilopterus princetonii* also indicates that the overall morphology of the paddle changed during ontogeny, with juveniles having podomere VI-9 much smaller in relation to VI-8 than in adults, and the serrations on podomeres VI-7 and VI-8 being less developed [5], although this cannot be confirmed in the currently available material of *Strobilopterus proteus*. The ‘*Carcinosoma*’ *scorpiodes* material also shows this trend [84], and this may be linked to earlier eurypterid instars being more active swimmers.

Another major difference in the earliest α instar of *Strobilopterus proteus* is the development of the posterolateral regions of the carapace being drawn out into long genal spines, a plesiomorphic characteristic that is usually considered absent in Sclerophorata (eurypterids and arachnids) but is present in xiphosurans and chasmataspidids [2]. The genal spines have been reduced to small posterolateral extensions of the carapace by stage β, with the first opisthosomal tergite shown to be fully laterally expressed behind the flattened carapace posterior margin. Similar posterolateral extensions are known from a number of other eurypterids, including *Eurypterus*[80], *Drepanopterus*[50], and *Adelophthalmus*[85], and it is possible that these, too, represent the vestigial remnants of genal spines. In the γ and δ instars the lateral portions of the second opisthosomal tergite expand anteriorly until they are overlapped by the carapace posterolateral extensions, the lateral portions of the first tergite apparently having been reduced.

The median ocelli are first observable in stage β specimens and are comparatively large and unpaired, set independently into the carapace cuticle without being positioned on a raised ocellar node. The ocelli are comparatively smaller in γ specimens but still situated independently on the carapace; however, in the δ instars the ocelli are now located together on a raised, cardioid ocellar node. The distinctive carapace cuticular ornamentation also changes throughout ontogeny, with the characteristic orientation of scales pointing away from the lateral eyes and angling around the carapace margins first being recognised in the γ specimens and becoming well-developed in δ individuals, the scales being elongated in the direction of orientation. The final definite trend observable during the ontogeny of *Strobilopterus proteus* is one of comparative shortening of the pretelson which is distinctly elongated in α individuals, the length of the segment being approximately twice the width. The length of the pretelson is gradually reduced through the β and γ stages, until the segment is approximately equal in length and width in δ instars.

The earliest ontogenetic stages of *Strobilopterus proteus* also hint at some trends that are not directly observable within the species. The metastoma is comparatively broad in the α specimens, being almost oval-shaped. The adult morphology of the metastoma in *Strobilopterus*, however, appears to be narrow, as suggested by *Strobilopterus princetonii* Tetlie [5]. As noted above, the metastoma of other eurypterid species has been observed to narrow comparatively as the individual matured, and this could explain the more oval morphology of the structure in the early instars of *Strobilopterus proteus*. These instars also preserve long hairs projecting from the margins of the opisthosomal opercula, with shorter hairs fringing the epimera. Hairs observed on disarticulated opercula of larger individuals are much shorter, often not projecting beyond the margin of the operculum, and it seems that these hairs, too, become comparatively reduced throughout development; a similar trend can be seen in early instars of *Limulus*[86]. Unlike *Limulus*, however, the earliest *Strobilopterus* instars possess the full adult complement of opisthosomal segments and appendages, and this suggests that true direct development may be another characteristic linking eurypterids and arachnids.

Finally, it is unusual that the type A genital appendages of the α instars are so large and well-developed, extending as far as they do to the anterior of the seventh opisthosomal segment. This phenomenon has been reported previously in eurypterids, with juveniles having comparatively enlarged genital appendages [78]. Such development of the sexual organs is generally a sign of sexual maturity, and in males of modern *Limulus* this only occurs in the final moult [81]. The holotype specimen of *Strobilopterus princetonii*, however, possesses a type A genital appendage that only extends down to the fourth opisthosomal segment and, while drawing comparisons between species can be difficult, it is possible that the genital appendage also became relatively shorter throughout ontogeny, a trend most unexpected for a sexual organ. Such a trend is, however, apparent in the prosomal appendages, which represent the endopods of biramous limbs in which the exopod has been reduced. The genital appendage has been suggested to comprise the fused endopods of the genital operculum, with the ala being formed from the exopods [87]. The apparently conflicting ontogenetic trend seen in the genital appendage therefore is due to the appendage not being the sexual organ itself but rather an ancillary structure that follows the development trend of the endopods from which it is derived, while the gonopores, which it overlies, may not fully develop until the final moult, as would be expected. This provides support for the hypothesis that the genital appendage represents a modified endopod of a biramous limb and shows the power of utilising ontogenetic pathways and trends in resolving homology statements.

### Implications of ontogenetic data for phylogenetic analyses

Prior to this study, the Cottonwood Canyon material now assigned to *Strobilopterus proteus* had been considered to represent a number of distinct species, with notes held alongside the specimens at the Field Museum even suggesting that one of the juvenile individuals (FMNH PE 6165) was an aglaspidid. As mentioned earlier, chelicerate palaeontologists have traditionally neglected to consider ontogeny when describing species, and so it is fully possible that a number of species actually represent juveniles. Given the increasing application of phylogenetic methodology in chelicerate palaeontology it is important to recognise whether including such ontogenetic species in phylogenetic analyses significantly perturbs the resulting topology relative to that retrieved utilising only adult instars. Often the variations in juvenile morphology appear to reflect primitive character states and, theoretically, juveniles coded as part of an analysis could clade with more primitive groups than their adult counterparts due to an assortment of primitive character states that are lost during later ontogeny – essentially a similar problem to that noted for paedomorphic species [88]. This could then cause further problems with the juvenile taxa introducing derived character states into basal clades and increasing attraction of disparate taxa in different clades, reducing branch support and potentially collapsing the inter-clade topology resulting in deep-level polytomies.

**Figure 28:**
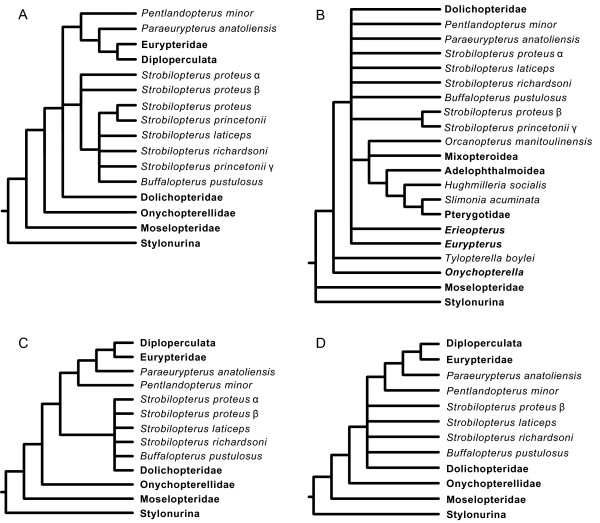
**Results of phylogeny experiments.** Strict consensus trees retrieved when including juvenile instars in the phylogenetic analysis. **A**: All *Strobilopterus proteus* and *Strobilopterus princetonii* instars included. **B**: Adult *Strobilopterus proteus* and *Strobilopterus princetonii* instars excluded, juvenile instars included. **C**: Only α and β *Strobilopterus proteus* instars included, all other *Strobilopterus proteus* and *Strobilopterus princetonii* instars excluded. **D**: Only the *Strobilopterus proteus* β instar included, all other *Strobilopterus proteus* and *Strobilopterus princetonii* instars excluded.

**Figure 29:**
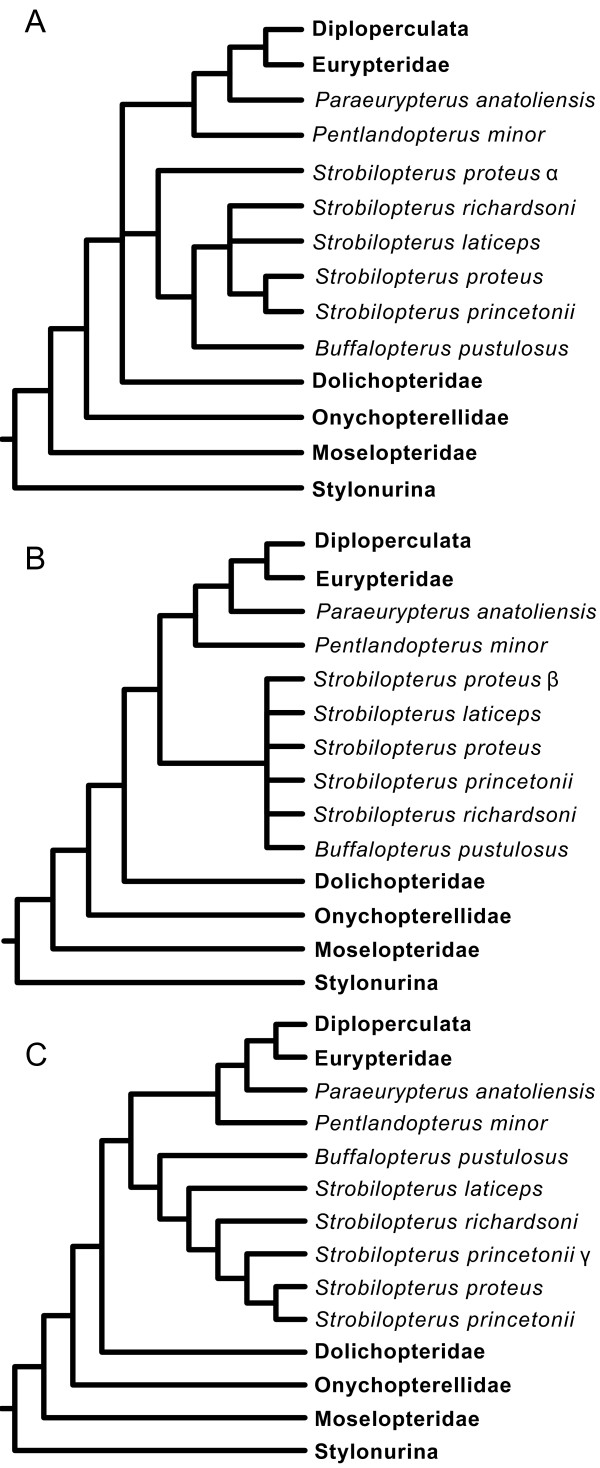
**Results of phylogeny experiments.** Strict consensus trees retrieved when including juvenile instars in the phylogenetic analysis. **A**: With the addition of the *Strobilopterus proteus* α instar. **B**: With the addition of the *Strobilopterus proteus* β instar. **C**: With the addition of the *Strobilopterus princetonii* γ instar.

In order to test whether this scenario holds true with the current phylogeny different juvenile ontogenetic stages of *Strobilopterus proteus* (α, β) and *Strobilopterus princetonii* (γ) were coded for the analysis and different permutations run with varying combinations of juvenile instars included, the results of which are shown here (Figures 28, 29). Performing the analysis with the inclusion of all juvenile instars alongside the original species codings results in a loss of resolution within the Strobilopteridae, with *Buffalopterus pustulosus*, *Strobilopterus richardsoni*, *Strobilopterus laticeps* and *Strobilopterus princetonii* γ forming a polytomy alongside a clade comprising *Strobilopterus proteus* and *Strobilopterus princetonii*. The two earliest instars, *Strobilopterus proteus* α and β, form a polytomy below the main strobilopterid clade. The broad-scale topology of the tree remains unchanged, although Dolichopteridae and Strobilopteridae now form a polytomy rather being fully resolved as part of the paraphyletic grade leading to Diploperculata (Figure 28A). The ensemble Consistency and Retention Indices are both lower than in the original analysis, being 0.388 and 0.778 respectively, resulting in a Rescaled Consistency Index of 0.302. Removing the original species codings so that only the juvenile instars are included in the analysis results in a widespread loss of resolution, with a large number of non-diploperculate Eurypterina forming a large polytomy with a number of smaller clades retained within it (Figure 28B). Furthermore, the monophyly of Eurypterina is also uncertain as moselopterids are resolved in a polytomy with Stylonurina and the other Eurypterina. Further removing *Strobilopterus princetonii* γ, so that only *Strobilopterus proteus* α and β remained, returns much of the tree to its original topology; however, the strobilopterids instead resolve in a basal polytomy as part of the dolichopterid clade (Figure 28C), a relationship that is still retained when removing *Strobilopterus proteus* β from the analysis. Including solely *Strobilopterus proteus* β or *Strobilopterus princetonii* γ results in both cases in a similar topology to the first analysis, however the strobilopterid clade is completely broken down and forms a polytomy with Dolichopteridae and the remaining Eurypterina (Figure 28D). Finally, including each of the earlier instars individually into the analysis results in *Strobilopterus proteus* α resolving at the base of Strobilopteridae, with a loss of resolution between *Strobilopterus laticeps* and *Strobilopterus richardsoni* along with Dolichopteridae and Strobilopteridea (Figure 29A), while *Strobilopterus proteus* β simply polytomies the entirety of Strobilopteridae while retaining them as a definite clade separate to dolichopterids (Figure 29B). *Strobilopterus princetonii* γ meanwhile resolves as the sister taxon to *Strobilopterus proteus* and *Strobilopterus princetonii* without altering the rest of the tree in any manner (Figure 29C).

These experiments are instructive in a number of ways. First, it appears that the earlier the instar the more basal within the clade it resolves. Second, the inclusion of instars can destabilise the internal topology of the clade resulting in its ground plan becoming uncertain or different altogether, and this can, in turn, result in loss of resolution over the analysis as a whole. Third, it would also seem that including ontogenetic species alongside more mature instars of the same species goes someway to conserving the tree topology. Even then, the presence of juveniles can be detrimental, interfering with metrics for assessing the completeness of the fossil record; if a group was particularly long-ranging a juvenile found in strata towards the end of its range but resolved at the base of the clade would decrease the values of results calculated using the Relative Completeness Index [89], Stratigraphic Consistency Index [90] and Gap Excess Ratio [91] metrics through an inferred ghost range of the juvenile taxon back to the origin of its group. If the juvenile were to resolve at the base of a larger clade, thereby possible affecting several nested clades, the influence on these metrics would be magnified. This, then, leads to incorrect assumptions regarding the completeness of the fossil record and the stratigraphical fit of a phylogenetic topology. Therefore, whenever possible, juvenile specimens should be excluded from phylogenies intending to ascertain inter-clade relationships or be used as part of a broader study; however, a broader analysis looking at taxa in a number of different groups both within and without Eurypterida is needed in order to test whether these observation are valid in a broader context or apply solely given the pattern of development seen in *Strobilopterus*. It should also be noted that ontogenetic data should not be completely excluded from phylogenetic analysis, and that when carefully integrated it has the potential to provide new information that may help resolve competing topologies. As an example, the juvenile specimen of *Strobilopterus princetonii* shows that the terminal podomere of the paddle is reduced in earlier instars in contrast to the larger podomere recognised in the adults. This is an important observation as a reduction of the terminal podomere of appendage VI defines part of the eurypterine tree, and the realisation that strobilopterids do in fact exhibit this reduction is one of the reasons that they have been able to be separated from dolichopterids.

## Conclusions

The new species of eurypterid described here from Cottonwood Canyon are a new and important source of data on the postembryological development of an extinct arthropod group. Trends that have been inferred in the few previous studies into eurypterid ontogeny [7-9] are corroborated by the new material, while new developmental phenomena are also described for the first time; the ontogeny of eurypterids appears to broadly parallel that of extant and extinct horseshoe crabs [10,81,86] with the major exception that eurypterids may hatch with their full complement of opisthosomal segments and appendages, thus being true direct developers like arachnids, and not hemianamorphic direct developers as in xiphosurans. Ontogenetic data can also be important for informing on homology statements and the observed development of the genital appendage in *Strobilopterus proteus* lends support to the hypothesis that the appendage represents a fused opisthosomal endopod [87].

The inclusion of these taxa into a growing phylogenetic framework provides further resolution of the basal Eurypterina. Previous chelicerate workers have commonly neglected to differentiate between juvenile and adult morphologies, and our experiments using the different *Strobilopterus* instars have shown how including juvenile individuals into an otherwise well-resolved phylogeny can destabilise it. It is integral that future workers account for ontogeny when describing species and selecting taxa for phylogenetic analysis; the preliminary results presented here suggest that coding juveniles as operational units within a phylogenetic analysis will produce unresolved, potentially spurious results. Ontogenetic data should not, however, be excluded without thought; rather, serious attempts should be made to successfully integrate ontogenetic data into phylogenetic analyses without resorting to coding instars as evolutionary individuals. The logical alternative of coding ontogenetic data as separate characters is also problematic, however, as heterochronic perturbations in the timing of development and maturities can make the recognition of homologous developmental stages difficult. Recent studies on trilobites have shown that the protaspid larval phase does not encompass the same developmental stages in all trilobites [92], casting doubt on the validity of the standard direct comparison between final stage protaspides. In order to account for these issues, it has been suggested that comparisons be made only when the entire ontogenetic series is taken into account [92], and recent work has attempted to characterise this both descriptively [93] and quantitatively [94] in a number of trilobite species. In many cases however the entire ontogenetic series will not be available for study, and although instars can be recognised as in the current study it is impossible to correlate these stages with certainty between species. It is possible that in these situations ontogenetic data can still be included in phylogenies through careful character selection and definition, however a definitive procedure is at present lacking. If ontogenetic data could successfully be incorporated into phylogenetic analysis it could potentially have great utility in resolving groups that are at least partially defined by characteristics present only in the larval phases, as in some crustaceans [95,96]. Furthermore, it is only through accurate handling of ontogenetic data that the affinities of taxa derived through paedomorphosis can be accurately determined phylogenetically. This is key to resolving conflicts in groups where paedomorphically derived species a commonplace, such as amphibians, where paedomorphic species have been shown to behave during analysis in a manner similar to the juvenile ontogenetic stages coded herein [88]. While this study does not present a full solution to the issue, it does suggest that incorporating well-constrained ontogenetic characters in a phylogenetic analysis may be a preferable solution to the potentially destabilising influence of juvenile instars being included as distinct operational units.

## Competing interests

The authors declare they have no competing interests.

## Author’s contributions

JCL conceived of the study, documented the specimens, undertook the morphological interpretations, processed the images, produced the figures, performed the phylogenetic analysis, and wrote the manuscript. PAS participated in the writing of the manuscript. Both authors have read and approved the final manuscript.

## Supplementary Material

Additional file 1 **Electronic Appendix.** Electronic appendix PDF including a full character list and matrix for the phylogenetic analysis. Click here for file
