# Supplementary material for: Babes in the wood – a unique window into sea scorpion ontogeny
Source: BMC Evol Biol. 2013 May 10;13:98. doi: 10.1186/1471-2148-13-98 (PMC3679797; doi:10.1186/1471-2148-13-98)
Supplement: Additional file 1 — Electronic Appendix. Electronic appendix PDF including a full character list and matrix for the phylogenetic analysis. [file 1471-2148-13-98-S1.pdf]

ELECTRONIC APPENDIX

This is the Electronic Appendix to the article

Babes in the wood – a unique window into sea scorpion ontogeny

by

James C. Lamsdell and Paul A. Selden

Comprising the morphological character list and character matrix used in the phylogenetic analysis

Character list

1. Angular projection on the anterior of the carapace: absent (0); present (1).
2. Anterior margin of carapace: unornamented (0); denticulate (1).
3. Carapace marginal rim: present (0); absent (1).
4. Carapace genal spines: present (0); absent (1).
5. Carapace shape: horseshoe or wide-horseshoe (0); semicircular (1); quadrate (2); trapezoidal (3); wide-rectangular (4); subquadrate (5); campanulate (6); parabolic (7); spatulate (8); triangular (9); long-rectangular (10).
6. Carapace genal facets: absent (0); present (1).
7. Angle of genal facets: low angle (0); high angle (1).
8. Lateral eye shape: crescentic (0); expanded (1).
9. Size of palpebral lobe: small (0); large (1); absent (2).
10. Lateral eye position: centrilateral (0); centrimesial (1); antelateral (2); central (3); antemesial (4).
11. Lateral eye abuts carapace margin: absent (0); present (1).
12. Lateral eyes associated with ophthalmic ridge: present (0); absent (1).
13. Suture on ventral plates: *Eurypterus*-type (0); *Hughmilleria*- or *Hallipterus*-type (1); *Megalograptus*-type (2); *Erieopterus*-type (3).
14. Transverse suture on ventral plates: present (0); absent (1).
15. Chelicerae: small (0); able to extend beyond marginal rim (1).
16. Form of cheliceral peduncle: approximately equal in length to fixed ramus (0); longer than fixed ramus (1).

17. Denticles on chela: absent (0); present, small, undifferentiated (1); present, large, differentiated (2).
18. Largest denticle medially on fixed ramus: erect (0); inclined (1); acute (2).
19. Angular distal teeth on both rami: absent (0); present (1).
20. Relative lengths of appendages II-V: increasing posteriorly (0); forward appendages enlarged (1).
21. Morphology of spines on prosomal appendages II-IV: reduced [length < width of podomere] (0); regular [length ≈ width of podomere] (1); enlarged [length > width of podomere] (2); absent (3).
22. Mobility of spines on appendages II–V: fixed (0); moveable (1).
23. Orientation of spines on appendages II–V: ventral (0); anterior (1).
24. Spines on appendages II–V: thickened and highly sclerotised absent (0); present (1).
25. Armature of anterior prosomal appendages flattened and laterally expanded into blades: absent (0); present (1).
26. Prosomal appendage podomeres thicken distally: absent (0); present (1).
27. Morphology of appendage V podomeres: undifferentriated and broad (0); podomeres tubular, lacking armature except for penultimate distal podomere (1).
28. Appendage V armature: non-spiniferous (0); spiniferous (1).
29. Coxal laden: absent (0); present (1).
30. Morphology of prosomal appendage VI: equal in width along length (0); distally expanded into swimming leg (1).
31. First podomere of prosomal appendage VI that fully projects beyond carapace margin: Fourth podomere (0); Sixth podomere (1); Third podomere (2).

32. Shape of proximal podomere of appendage VI: narrow [L/W≥2.0] (0); expanded [L/W<2.0] (1).
33. Anterior margin of coxa VI undifferentiated (0); expanded to form 'ear' (1).
34. Shape of 'ear' on coxa VI: triangular (0); rectangular (1); subquadrate/semicircular (2).
35. Anterior denticle of coxa VI large: absent (0); present (1).
36. Angle between VI-3 and VI-4: 180º (0); not 180º (1).
37. Length of podomeres VI-4 and VI-5: VI-5>VI-4 (0); VI-4≥VI-5 (1).
38. Length of podomeres VI-4 when VI-5 is not longer: >VI-3 and VI-5 (0); equal to VI-3 and VI-5 (1).
39. Podomere VI-5 bordering podomere VI-7: absent (0); present (1).
40. Distal podomere margin of VI-6 modified: absent (0); present (1).
41. VI-7 lateral margins: unornamented (0); enlarged serrations (1); small serrations (2).
42. Angular projection on the anterior of podomere VI-7: absent (0); present (1).
43. Podomere 7a on appendage VI: absent (0); present on posterior of appendage (1); present on anterior of appendage (2).
44. Width of posterior VI-7a: narrow [less than 50% width of VI-7] (0); wide [more than 50%] (1).
45. Shape of posterior VI-7a: oval (0); triangular (1).
46. VI-8 lateral margins: unornamented (0); small serrations (1); enlarged serrations (2).
47. VI-8 anterior projection: absent (0); present (1).
48. Additional moveable lobe on VI-8: absent (0); present (1).
49. Morphology of VI-8/VI-9 joint: joint flush (0); VI-9 set into VI-8 (1).

50. Length of VI-9 (as ratio of VI-8): large [>25% of VI-8 length] (0); small [<25% of VI-8 length] (1); totally absent (2).
51. Shape of podomere VI-9: spinose (0); triangular, pentagonal or oval (1).
52. VI-9 margin: no ornament (0); serrated (1).
53. VI-9 position: centrally on podomere 8 (0); migrating towards posterior of podomere 8 (1).
54. VI-9 expanded: absent (0); present (1).
55. Podomere cuticular morphology: rounded (0); ridged (1).
56. Metastoma anterior: smooth or shallow notch (0); deeply notched (1).
57. Shape of posterior margin of metastoma: rounded (0); truncated/flattened (1); angular (2); notched (3).
58. Metastoma with posterior median cleft: absent (0); present (1).
59. Metastoma shape: broad (0); petaloid, markedly narrower in width (1).
60. First segment expressed dorsally: expressed (0); folded under carapace (1).
61. Microtergite: absent (0); present (1).
62. Genital operculum morphology: consisting of three visible segments (0); consisting of two visible segments (1); unfused (2).
63. Morphology of anterior opercular plate: fully expressed (0); lateral expression only (1); completely supressed (2).
64. Thickness of anterior opercular plate: fully expressed (0); narrow (1).
65. MOP and POP morphology: separate/with clear suture (0); without suture but with ornament differentiation (1); without suture and without ornament differentiation (2).

66. Unsegmented genital appendages: absent (0); present (1).

67. Type-A genital appendage spoon-shaped: absent (0); present (1).

68. Type-A genital appendage with median, distal indentation: absent (0); present (1).

69. Type-B genital appendage oval: absent (0); present (1).

70. Morphology of type A genital appendage furca: fused (0); unfused (1).

71. Spatulae on type A genital appendage: absent (0); present (1).

72. Morphology of spatulae: angular (0); rounded (1).

73. Moveable preabdominal spines: absent (0); present (1).

74. Postabdominal epimera: present (0); absent (1).

75. Preabdominal/postabdominal boundary: between 7th and 8th segments (0); between 6th and 7th segments (1); between 4th and 5th segments (2).

76. Epimera on segment 7: absent (0); present (1).

77. Number of segments in postabdomen: 3 (0); 5 or 6 (1); 9 (2).

78. Postabdomen: undifferentiated (0); narrowing from preabdomen (1).

79. Caudal postabdoment: absent (0); present (1).

80. Posterior margin of all opisthosomal tergites crenulate: absent (0); present (1).

81. Posterior margin of segments 7-9: smooth (0); dentate (1).

82. Posterior margin of segments 10-12: smooth (0); dentate (1).

83. Epimera on pretelson: absent (0); angular (1); rounded (2); quadrate (3).

84. Laterally expanded pretelson: absent (0); present (1).

85. Median carina on pretelson: absent (0); present (1).

86. Telson shape: straight (0); curved (1); laterally expanded (2).

87. Laterally expanded telson shape: rounded (0); triangular (1).

88. Telson base: flattened (0); bulbous expansion (1).
89. Telson bilobed: absent (0); present (1).
90. Median carina on telson: absent (0); low carina (1); vertical rudder (2).
91. Telson laterally ornamented with scales: absent (0); present (1).
92. Telson margin: smooth (0); serrated (1).
93. Opisthosoma lateral division: none (0); trilobed (1).
94. Primary opisthosomal ornamentation: scales (0); closely spaced pustules (1); coarse pustules (2).
95. Primary carapace ornamentation: scales (0); closely spaced pustules (1); coarse pustules (2).
96. Principal scale on carapace: absent (0); present (1).
97. Carapace ornament including elongate pustules that angle away from the lateral eyes and curve around the carapace margin: absent (0); present (1).
98. Row of node-like scales on dorsal opisthosomal segments: absent (0); present (1).
99. Ornamentation of angular scales across posterior of tergite segments: absent (0); present (1).
100. Longitudinal rows of angular scales on tergites: absent (0); present (1).
101. Ornament of chevron scales: absent (0); present (1).
102. Ornament of linguoid scales: absent (0); present (1).
103. Form of posterior margin of articulating facet on tergites: row of pustules (0); row of scales (1); groove (2).

104. Genital operculum striate ornament marked by highly sclerotised, broad lunate scales: ornament independent of sclerotised scales (0); ornament congruent with broad, sclerotised lunate scales (1).

Character matrix (main analysis)

(A = [0,1]; B = [1,2])

Taxon		10		20		30		40		50		60		70		80		90		100	
Weinbergina opitzi	00100	0-0-?	00310	00--0	11000	00000	010-0	00-00	000--	00000	00000	----0	022-0	-----	--000	00000	00000	0-000	00011	00000	0000
Chasmataspis laurencii	00000	0-101	01???	?????	?????	0???0	?00-0	00-00	000--	000--	----0	????0	1????	?????	??002	02110	00000	0-000	00122	00100	00??
Octoberaspis ushakovi	00003	0-000	00???	?????	?????	000?1	0????	???0?	002--	00002	----0	02000	12000	00010	0-002	02100	00100	0-000	00022	00000	00?0
Diploaspis casteri	00003	0-100	001??	?????	00000	0???1	0????	??00	002--	00002	----0	????0	1200?	?????	??002	02100	00000	20000	01022	00100	00??
Loganamaraspis dunlopi	00003	0-?0?	??310	00--?	?????	0??00	000-?	0????	?????	?????	????0	02000	12000	000?0	0-012	02100	00000	0-?00	0?0--	-0000	00??
Brachyopterus stubblefieldi	00018	0-001	01?0?	????0	00000	000?0	000-?	00-00	000--	00000	00001	0???1	0????	0????	??0?0	01000	00000	0-000	000--	-0000	00??
Rhenopterus diensti	00017	0-001	01000	00--0	00000	00000	000-0	00-00	000--	00000	00001	00001	00000	0??00	0-001	01110	00000	1-000	00000	00000	0000
Parastylonurus ornatus	00010	0-000	01000	00--0	11000	00000	200-0	00-00	000--	00000	00000	11011	00000	00000	10000	01101	00100	0-001	00000	00000	00?0
Laurieipterus elegans	01018	0-000	0110?	????0	10000	00000	200-0	00-00	000--	000??	????0	1100?	0????	?????	??0??	?????	?????	?????	??000	00000	00?0
Kokomopterus longicaudatus	00015	0-001	01???	????0	11000	10100	000-0	00-00	000--	00000	00000	1100?	0????	???0?	??010	01101	00000	0-000	00111	00000	01?0
Hardieopteurs macrophthalmus	00010	0-001	01000	00--0	?????	10100	000-0	00-00	000--	00000	00000	1100?	0????	?????	??000	01101	00300	0-000	00111	00000	01?0
Drepanopterus pentlandicus	00010	10001	01000	00--0	1A001	10000	000-0	00-00	000--	00000	00000	11101	00000	?001?	10010	01100	00000	0-000	00011	00000	0000
Hibbertopterus scouleri	00010	11003	01000	00--0	2A001	10010	000-0	00-00	000--	000??	????0	1110?	0????	???1?	??010	01001	00000	0-000	00000	00000	01?0
Megarachne servinei	00013	0-003	01???	?????	2A001	1001?	?00-0	?????	?????	?????	????0	?????	0????	?????	?????	?????	?????	?????	???00	00???	????
Stoermeropterus conicus	00010	0-001	01110	00--0	00000	00000	01100	00-00	00100	00000	00001	10001	00010	00010	10100	01000	01B00	0-100	00011	00000	0000
Stoermeropterus latus	00010	0-001	01110	00--?	?????	0???0	0???0	00-00	00100	00000	00001	????1	00010	000??	11100	01000	01100	0-100	00000	00000	00?0
Stoermeropterus nodosus	00010	0-001	01???	?????	?????	0????	?????	?????	?????	?????	?????	10001	00010	000?0	11100	01000	0?200	0-100	00011	00100	00??

Vinetopterus struvei	01010	0-001	01310	00--0	00000	00000	01100	00-00	00100	00000	00001	00001	00010	0??1?	??000	01100	11000	0-?00	00011	00100	0000
Vinetopterus martini	01010	0-?01	0131?	?????	00000	00000	0????	00-00	00100	00000	00001	????1	0????	?????	??000	01100	11000	0-?00	00011	00100	00??
Moselopterus ancylotelson	00010	0-001	01310	00--0	10000	00000	01100	00-00	00100	00000	00001	00001	00010	0001?	10010	01100	11000	1-000	01011	00100	0000
Moselopterus elongatus	00010	0-001	01310	00--0	10000	00000	01100	00-00	00100	00000	00001	00001	00010	???1?	??010	01100	11000	1-000	01011	00?00	0000
Onychopterella augusti	00015	0-?01	01???	????0	11000	0??01	01100	00-00	00101	00010	00000	???0?	0????	?????	0-000	01100	00000	0-100	00000	0?000	00??
Onychopterella kokomoensis	00015	0-101	01310	00--0	11000	00101	01???	00-00	00101	00010	00000	00001	00010	000?1	0-000	01100	00200	0-100	00000	0?000	00?0
Dolichopterus macrocheirus	00012	0-012	01010	00--0	21000	00101	01110	11001	11101	20110	11010	11011	00110	000?0	10000	01100	00100	0-000	00000	00000	0000
Eurypterus remipes	00013	10101	01010	00--0	11000	01101	01120	01001	00111	10011	10000	00001	00110	00001	0-010	01100	00000	0-000	01000	10011	0010
Hughmilleria socialis	00017	0-122	11111	00--0	11000	00101	01120	01101	00111	00011	10100	0100?	012-2	00010	0-010	11100	00000	0-000	00000	00000	11?0
Tylopterella boylei	00015	0-101	01???	?????	?????	?????	?????	?????	?????	?????	?????	?????	0????	?????	??001	01100	00000	0-000	00000	00000	00??
Megalograptus ohioensis	01012	10104	01210	00--1	21110	01101	01120	01001	00111	00012	----0	03001	012-1	00000	0-011	01100	00000	0-000	00100	00011	0010
Mixopterus kiaeri	00012	10104	01?10	00--1	21110	00101	01120	01111	00111	00011	10000	0000?	012-2	000?0	0-010	01110	00000	1-000	00100	00000	00?0
Strobilopterus laticeps	00011	0-011	01???	?????	?????	????1	1????	01101	00111	00-11	10000	1101?	?0110	000?0	10???	????0	?????	?????	????1	0????	???1
Strobilopterus princetonii	00011	10013	01??0	00--0	10000	0??01	11120	01101	10111	20110	11000	1?01?	0???0	000?0	10000	11100	00000	?????	??011	0?0?0	0001
Strobilopterus richardsoni	00011	0-013	0131?	?????	?????	????1	?????	?????	10111	20011	10000	?????	?????	?????	?????	?????	?????	?????	??011	010??	00??
Strobilopterus proteus	00011	10013	0131?	????0	10000	00101	111?0	01101	10111	20110	11000	1001?	0???0	00010	10000	11100	00000	?????	??011	01010	0001
Buffalopterus pustulosus	00011	0-011	0131?	?????	?????	????1	1????	?????	?????	?????	?????	????1	00110	000??	0-000	01100	00100	20000	01011	01010	0000
Erieopterus microphthalmus	00014	11001	01310	00--0	10000	01101	01120	01001	00111	00011	00000	01001	00110	000?1	0-010	01100	00100	1-000	00000	00000	00?0
Paraeurypterus anatoliensis	00012	10011	01?1?	?????	11000	0???1	0????	010??	?????	?????	????0	????1	0????	?????	??0?0	0???0	?????	?????	??001	00010	001?
Pentlandopterus minor	00012	10011	01???	?????	?????	????1	0????	010??	?????	?????	?????	?????	0????	?????	??010	01100	00000	0-000	00011	00000	000?
Dolichopterus jewetti	00012	0-012	0???0	00--0	21000	00101	01110	11001	11101	20110	11010	11001	00110	000?0	10000	01100	00100	0-000	00001	00?00	0000
Ruedemannipterus stylonuroides	10016	0-012	01???	?????	?????	????1	0????	11001	?1101	?0110	10110	?????	0????	?????	??0??	?????	?????	?????	??011	00000	00??
Clarkeipterus testudineus	10002	0-012	01???	?????	?????	????1	0????	11001	?1101	?0?10	10010	?????	?????	?????	?????	?????	?????	?????	????1	00?00	????

Clarkeipterus otisius	10002	0-012	01???	?????	?????	?????	?????	?????	?????	?????	?????	?????	?????	?????	?????	?????	?????	?????	????1	00???	????
Erieopterus eriensis	00011	11001	0131?	????0	10000	0??01	01120	01001	00111	00011	00000	????1	00110	?????	??010	01100	00???	0-000	00000	00000	00??
Eurypterus henkeni	00013	10101	0101?	?????	11000	0??01	01120	01001	00111	00011	10000	0000?	0????	?????	0-010	01100	00100	0-000	01000	10011	0010
Eurypterus tetragonophthalmus	00013	10101	01010	00--0	11000	01101	01120	01001	00111	10011	10000	00001	00110	00001	0-010	01100	00200	0-000	01000	10011	0010
Eurypterus leopoldi	00013	10101	01???	?????	?????	????1	?????	?????	?0111	10011	10000	1000?	?????	?????	???0?	???00	00200	0-000	01?00	10011	00??
Eurypterus pittsfordensis	00013	10101	01010	00--0	11000	01101	01120	01001	00111	10011	10000	1000?	0????	?????	??000	01100	00200	0-000	01000	10011	001?
Eurypterus henningsmoeni	00013	10101	0101?	????0	?????	?1101	01120	01001	00111	10011	10000	0000?	0????	?????	0-010	01100	00200	0-000	01000	10011	001?
Orcanopterus manitoulinensis	10017	0-102	01??0	00--0	21000	001?1	0????	0??0?	00111	00010	10010	1100?	012-1	0??0?	??010	11100	00000	0-000	00000	00000	10?0
Pterygotus anglicus	00113	0-122	11111	12000	3----	00101	01121	01101	20111	10011	10100	0000?	012-2	11000	0-000	01100	00011	2000?	11000	00000	1110
Adelophthalmus sievertsi	00017	0-101	01110	00--0	3----	00101	01120	01101	00111	11011	10100	0000?	012-2	00000	10010	01100	00000	0-000	00000	00000	1120
Eurypterus dekayi	00013	10101	01010	00--0	11000	01101	01120	01001	00111	00011	10000	0000?	00110	000?1	0-010	01100	00200	0-000	00000	00010	0010
Carcinosoma newlini	00019	0-102	01?10	00--1	21110	00101	01120	01111	00111	10011	11010	0000?	012-2	000?0	0-010	01110	00000	0-000	00000	00000	00?0
Slimonia acuminata	00010	0-122	11??1	00--0	01000	00101	01120	01101	00111	00011	10100	1001?	012-2	00000	0-010	01100	00000	20000	11000	00000	11?0
Acutiramus bohemicus	??1??	?????	????1	1221?	3----	0??01	01121	01101	?????	?????	????0	1????	?????	110??	?????	?????	???11	20001	?1?0?	?????	11??
Acutiramus macrophthalmus	00115	0-122	11111	12210	3----	00001	01121	01101	20111	10011	10100	1100?	012-2	1110?	0-000	01100	00011	20001	11000	00000	1110
Erettopterus bilobus	00112	0-122	11111	11000	3----	0??01	01120	01101	20111	10011	10100	1000?	012-2	1101?	0-000	01100	00011	20012	11000	00000	1110
Erettopterus osiliensis	00112	0-122	11??1	1200?	3----	0??01	01120	0??01	20111	10011	10100	1000?	012-2	1111?	0-000	01100	00011	20012	11000	00000	11?0
Erettopterus serricaudatus	?????	?????	????1	1100?	?????	?????	?????	?????	?????	?????	?????	1000?	?????	100??	?????	?????	???1?	20012	11?0?	?????	11??
Erettopterus waylandsmithi	00112	0-122	11??1	1200?	?????	?????	?????	?????	?????	?????	?????	?????	?????	?????	?????	?????	?????	20?1?	???00	00???	11??
Jaekelopterus rhenaniae	00113	0-122	11??1	1210?	3----	00001	01121	?????	?????	?????	????0	1100?	012-2	11100	0-000	01100	00011	21002	10000	00000	1110
Jaekelopterus howelli	?????	?????	????1	1210?	?????	???01	?1121	?????	?????	10011	10100	1100?	?12-2	11000	0-???	???00	?0011	21002	11?0?	?????	1110
Ciurcopterus ventricosus	00104	0-122	11???	????0	01000	00101	01???	01101	00111	?????	????0	?????	012-2	100?0	0-000	01100	00010	20000	10000	00000	11?0
Nanahughmilleria norvegica	00?17	0-101	01110	????0	11000	00101	01120	01101	00111	11011	10100	1000?	012-2	????0	10000	11100	000??	0?0??	?0000	00000	1110

Character matrix addition (juvenile instars)

Instar		10		20		30		40		50		60		70		80		90		100	
Strobilopterus proteus α	00001	0-011	0131?	????0	10000	0010?	011?0	0110?	?????	?????	????0	1001?	0???0	000?0	10000	11100	00000	?????	??011	00000	000?
Strobilopterus proteus β	00011	10013	01???	????0	10000	0??01	011??	01101	?????	?????	????0	?????	0????	?????	??000	11100	00000	?????	??011	00000	000?
Strobilopterus princetonii γ	00011	10013	01???	????0	10000	0???1	1????	???01	10111	10011	10000	?????	0????	?????	??000	11100	00000	?????	??011	0?0?0	000?
